# Transcriptomics identifies key defense mechanisms in rice resistant to both leaf-feeding and phloem feeding herbivores

**DOI:** 10.1186/s12870-021-03068-5

**Published:** 2021-06-30

**Authors:** Yi Li, Boon Huat Cheah, Yu-Fu Fang, Yun-Hung Kuang, Shau-Ching Lin, Chung-Ta Liao, Shou-Horng Huang, Ya-Fen Lin, Wen-Po Chuang

**Affiliations:** 1 Department of Agronomy, National Taiwan University, Taipei, 10617 Taiwan; 2 Crop Environment Section, Taichung District Agricultural Research and Extension Station, COA, Changhua Country, 51544 Taiwan; 3 Department of Plant Protection, Chiayi Agricultural Experiment Station, Taiwan Agricultural Research Institute, Council of Agriculture, Chiayi, 60044 Taiwan

**Keywords:** *Oryza sativa*, *Cnaphalocrocis medinalis*, *Nilaparvata lugens*, RNA-Seq, Dual resistance

## Abstract

**Background:**

Outbreaks of insect pests in paddy fields cause heavy losses in global rice yield annually, a threat projected to be aggravated by ongoing climate warming. Although significant progress has been made in the screening and cloning of insect resistance genes in rice germplasm and their introgression into modern cultivars, improved rice resistance is only effective against either chewing or phloem-feeding insects.

**Results:**

In this study, the results from standard and modified seedbox screening, settlement preference and honeydew excretion tests consistently showed that Qingliu, a previously known leaffolder-resistant rice variety, is also moderately resistant to brown planthopper (BPH). High-throughput RNA sequencing showed a higher number of differentially expressed genes (DEGs) at the infestation site, with 2720 DEGs in leaves vs 181 DEGs in sheaths for leaffolder herbivory and 450 DEGs in sheaths vs 212 DEGs in leaves for BPH infestation. The leaf-specific transcriptome revealed that Qingliu responds to leaffolder feeding by activating jasmonic acid biosynthesis genes and genes regulating the shikimate and phenylpropanoid pathways that are essential for the biosynthesis of salicylic acid, melatonin, flavonoids and lignin defensive compounds. The sheath-specific transcriptome revealed that Qingliu responds to BPH infestation by inducing salicylic acid-responsive genes and those controlling cellular signaling cascades. Taken together these genes could play a role in triggering defense mechanisms such as cell wall modifications and cuticular wax formation.

**Conclusions:**

This study highlighted the key defensive responses of a rarely observed rice variety Qingliu that has resistance to attacks by two different feeding guilds of herbivores. The leaffolders are leaf-feeder while the BPHs are phloem feeders, consequently Qingliu is considered to have dual resistance. Although the defense responses of Qingliu to both insect pest types appear largely dissimilar, the phenylpropanoid pathway (or more specifically phenylalanine ammonia-lyase genes) could be a convergent upstream pathway. However, this possibility requires further studies. This information is valuable for breeding programs aiming to generate broad spectrum insect resistance in rice cultivars.

**Supplementary Information:**

The online version contains supplementary material available at 10.1186/s12870-021-03068-5.

## Background

Rice (*Oryza sativa* L.) is cultivated on 160 million hectares of land in over a hundred countries, and approximately 491 million tons of milled rice are produced annually [1]. Rice is the most important staple food for half of the world’s population, especially in Asia and Africa [2]. However, insect pests pose a long-standing threat to rice farming worldwide [3]. There are over 100 species of rice-feeding insect pests, of which approximately 20 species are considered major pests that severely affect rice yield [4]. Recently, frequent and widespread outbreaks of major insect pests of rice, including the brown planthopper (BPH; *Nilaparvata lugens*) and rice leaffolder (*Cnaphalocrocis medinalis*), have caused losses of hundreds of millions of dollars annually and threatened food security [3, 5]. Insect pests cause severe damage to rice plants, as these pests thrive in the warm and humid environment of fertile paddy fields [6]. Moreover, insect pest infestations in paddy fields are predicted to become more rampant and serious in a warming climate that increases the metabolic rates and population growth of insects [7].

For over 50 years, spraying chemical insecticides has been the main strategy for managing crop pests [8]. However, the misuse of insecticides compromises the food safety and disrupts ecological and environmental integrity, which could lead to secondary pest outbreaks [9]. Therefore, policy makers in different countries are beginning to adopt environmentally friendly pest management strategies to minimize insecticide use [10]. For example, integrated pest management (IPM) is a long-term, environmentally friendly strategy that aims to control pests through a combination of approaches such as cultural practices, biological control and the use of resistant varieties [11]. Regarding the latter, a concerted effort has been made over the past decades to screen for insect resistance in rice germplasm, identify resistance genes, characterize the molecular mechanisms of host resistance, and introgress the resistance genes into modern cultivars [12]. To date, 34 resistance loci against the BPH have been identified in rice germplasm, of which 14 loci have been cloned and 5 loci have been functionally characterized (*Bph3*, *Bph14*, *Bph18*, *Bph26* and *Bph29*), while fewer than 10 genes have been introgressed into modern rice cultivars [12, 13].

Rice-feeding insect pests have different feeding guilds, but they can generally be divided into two groups: chewing insects and piercing-sucking insects [14]. Chewing insects, such as leaffolders and stem borers, have mouthparts that tear off and chew the foliage, causing extensive mechanical wounding to host plants [15]. Mechanical wounding prompts jasmonic acid (JA) production and signaling pathways, activating defense mechanisms such as the oxidative burst, induction of JA-responsive genes and generation of trypsin protease inhibitors that retard the feeding and growth of chewing insects [12]. On the other hand, piercing-sucking insect pests, such as planthoppers and leafhoppers, have specialized slender, tapering stylet mouthparts that penetrate the apoplastic space of epidermal cells and suck phloem sap [16]. Hoppers are also vectors of plant viruses, causing secondary damage to plants [17]. Previous studies indicated that salicylic acid (SA) biosynthesis and signaling are induced in rice plants in response to feeding by the BPH [18, 19].

Several studies of mutant rice lines defective in JA biosynthesis and signaling have confirmed the involvement of different phytohormone pathways in their defenses against chewing and phloem-feeding insects [20]. For example, knockout of a JA-biosynthetic lipoxygenase gene (*LOX*) increased the susceptibility of rice plants to striped stem borers (chewing insects), but improved their resistance to BPHs (phloem-feeding insects), presumably due to decreased JA and increased SA and hydrogen peroxide levels [21]. In addition, knockout of the coronatine-insensitive 1 gene (*COI1*; encodes JA receptor) increased the susceptibility of rice plants to chewing insects but did not alter rice resistance/susceptibility to BPHs, implying that JA signaling is not implicated in the rice response to the BPH [22]. Taken together, the antagonistic crosstalk between JA and SA pathways is a factor compromising the identification of plants with dual resistance to chewing and piercing-sucking insects [23].

Previous studies consistently showed that the Qingliu (QL) rice variety is resistant to leaffolder infestation [24–26]. Qingliu is a local native indica rice variety in Taiwan where its cultivation record can be dated back to the Qing dynasty era around 1742 [27]. In the past, this native variety was grown on a large scale because of its short life cycle, high yield and seed quality [27]. However, given that Qingliu is excessively tall and tends to lodge, its cultivation was eventually phased out in favor of modern semidwarf rice varieties developed during the Green Revolution [27]. Recently, a comparative proteomic study showed that the enzymes involved in flavonoid and JA biosynthesis were expressed at higher levels in Qingliu than in the susceptible check Taichung Native 1 (TN1) before and during leaffolder herbivory, potentially contributing to Qingliu resistance to chewing insects [28]. However, the RNA sequencing (RNA-Seq) profiles of Qingliu in response to leaffolder herbivory are not yet available. The objectives of this study are to (i) assess whether Qingliu has resistance to phloem-feeding BPHs and (ii) highlight the transcriptomic changes in key defense-related processes in Qingliu subjected to leaffolder and BPH infestation.

## Results

### Qingliu is moderately resistant to brown planthopper infestation

**Fig. 1:**
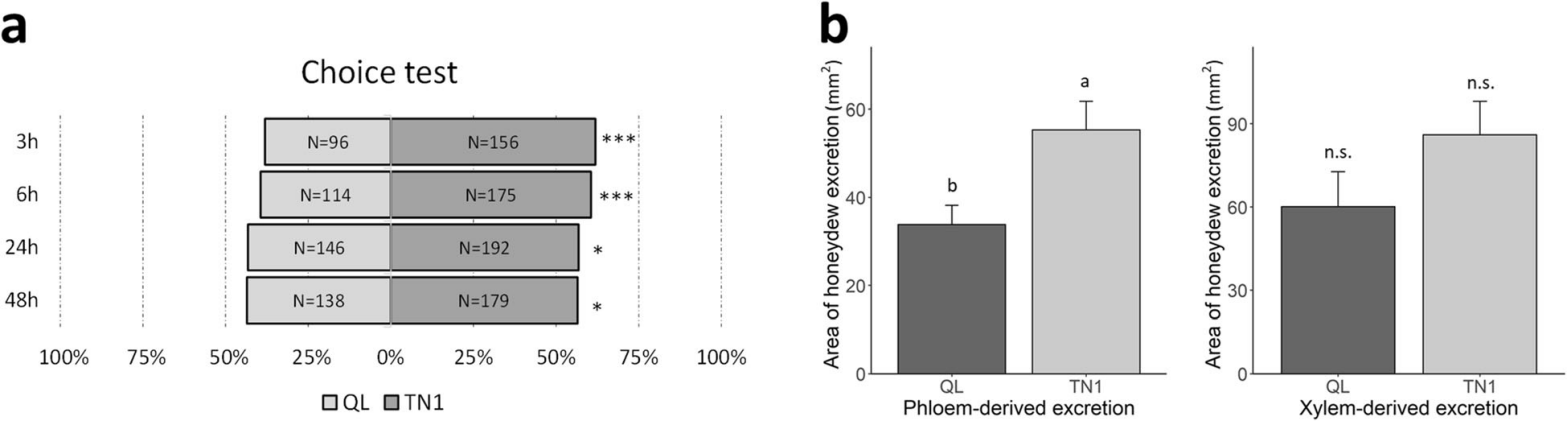
Evaluation of Qingliu resistance to BPH. **a** Comparison of settlement preference of third instar *N. lugens* nymphs between Qingliu and TN1 at 3, 6, 24 and 48 h of infestation (*N* = 400). Comparison of the area of **b** phloem-derived and xylem-derived honeydew excretion between gravid female *N. lugens* adults feeding on Qingliu and TN1. The experiment was repeated three times with a sample size of 8–10 (mean area ± SE). The settlement preference was analyzed using the *z*-test (*p < 0.05; ***p < 0.001), whereas the honeydew excretion test was analyzed using two-way ANOVA and LSD post hoc test with different letters denoting significant differences (*p* < 0.05). QL = Qingliu, n.s. = not significant

The standard seedbox screening test (SSST) showed that Qingliu was relatively less severely infested by BPH (average damage score = 5.0) than the two susceptible cultivars TN1 (8.3) and TNG67 (7.0) (two-way ANOVA and LSD post hoc test, *p*-value < 0.001) (Table 1). In addition, Qingliu also had the same average damage score as two moderately resistant checks: Mudgo and H105. As expected, Baiqiaowan, a leaffolder-resistant check, was highly susceptible to BPH infestation (9.0) (Table 1). In addition, the modified seedbox screening test (MSST) revealed a consistent result with the SSST (two-way ANOVA and LSD post hoc test, *p*-value < 0.001) (Table 1). These results indicated that Qingliu is moderately resistant to BPH infestation. In the choice test between Qingliu and TN1, a lower number of BPHs settled on Qingliu than on TN1 after 3, 6, 24 and 48 h of insect treatment (*z*-test, *p*-value = 0.0002, 0.0003, 0.0124, 0.0213 at the four time points) (Fig. 1a).

**Table 1:** Evaluation of Qingliu resistance to *N. lugens* using SSST and MSST

Standard seedbox screening test (SSST)						
Variety	Mudgo	Qingliu	H105	TN1	TNG67	Baiqiaowan
Damage score	5.0 ± 0.0c	5.0 ± 0.0c	5.0 ± 0.0c	8.3 ± 1.2a	7.0 ± 0.0b	9.0 ± 0.0a
*p*-value	2.37E-07					
**Modified seedbox screening test (MSST)**						
Variety	Mudgo	Qingliu	H105	TN1	TNG67	Baiqiaowan
Damage score	5.0 ± 0.0c	5.0 ± 0.0c	5.7 ± 1.2bc	7.0 ± 0.0ab	8.3 ± 1.2a	8.3 ± 1.2a
*p*-value	0.000388					

Two BPH-moderately resistant checks: Mudgo and H105. Two BPH-susceptible checks: TN1 and TNG67. The average damage score ± SD from three experimental repeats was analyzed with two-way ANOVA and LSD post hoc test with different letters denoting a significant difference (*p* < 0.05)

In the honeydew excretion test, individual BPHs feeding on Qingliu excreted a lower volume of phloem-derived excretion than those feeding on TN1 (two-way ANOVA, *p*-value = 0.0100) (Fig. 1b). This suggests that BPHs feeding on Qingliu either had less access to phloem sap or lower phloem sap intake rate than those feeding on TN1. Moreover, individual BPHs feeding on either Qingliu or TN1 excreted a similar volume of xylem-derived excretions (two-way ANOVA, *p*-value = 0.1443) (Fig. 1b). This implies that the BPHs feeding on Qingliu and TN1 either had equal access to xylem sap or similar xylem sap intake rates.

### A greater transcriptional change occurs at the infestation site of Qingliu than in other tissues in response to leaffolder and brown planthopper infestation

To discover the molecular responses of Qingliu potentially conferring the observed dual resistance to chewing and piercing-sucking insects, RNA-Seq profiles of the leaf and sheath tissues were generated under uninfested control, leaffolder-infested (24 h) and BPH-infested (24 h) conditions. A total of thirty RNA-Seq samples were generated with five biological replicates in each experimental group (2 tissues × 3 conditions × 5 biological replicates). RNA-Seq produced a large number of raw reads per sample, falling within the range of 39,700,000-52,700,000 (Table S1). A total of 36,000,000-49,200,000 clean reads per sample (90–93% of raw reads) passed the read trimming and filtering steps (Table S1). At least 92.71% of the clean reads per sample were mapped to the rice reference genome with at least 89.16% of clean reads mapped to a single genomic locus, confirming the quality of the RNA-Seq samples (i.e., free of microbial contamination) (Table S1).

Principal component analysis (PCA) was carried out to assess the homogeneity of the biological replicates (Fig. S1). At principal component 1 (13.25%), which showed the largest variation among the RNA-Seq samples, all fifteen leaf samples were segregated from the fifteen sheath samples. The second largest variation among the RNA-Seq samples (7.66%) was contributed by the relatively high heterogeneity among the sheath samples compared with that of the leaf samples. In each tissue, the samples of three different experimental conditions did not segregate considerably, implying the mild effect of both insect infestations on transcriptomic changes in Qingliu. Nevertheless, the biological replicates of each experimental group formed their own clusters (Fig. S1).

**Fig. 2:**
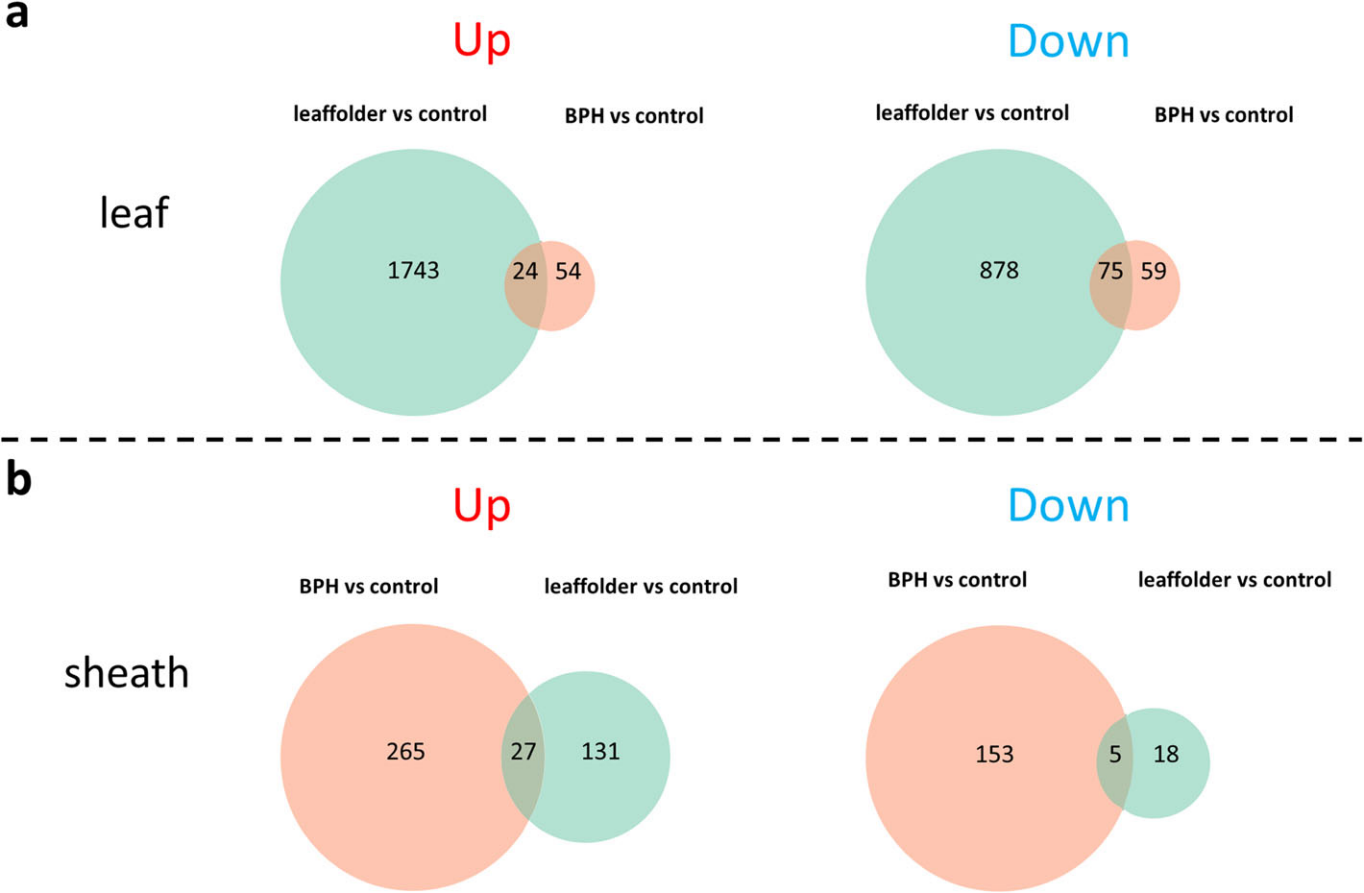
The number of differentially expressed genes identified in Qingliu under leaffolder and BPH infestation. The differentially expressed genes are shown as up- and downregulated genes **a** in leaves and **b** in sheaths. Up = upregulation and down = downregulation

At 24 h of leaffolder infestation, a higher number of DEGs (2720 vs 181 DEGs) were identified in the leaves than in the sheaths (Fig. 2; Table S2a, b). This finding was expected because the leaf is the infestation site of leaffolders. Among the DEGs in leaves, 1767 genes (64.96%) were upregulated and 953 genes (35.04%) were downregulated (Fig. 2; Table S2a). In contrast, more DEGs were detected in the sheaths than in the leaves at 24 h of BPH infestation: 450 vs 212 DEGs (Fig. 2; Table S2c, d). This was also expected because the sheath is the infestation site of the BPH. Among the DEGs in sheaths, 292 genes (64.89%) were upregulated and 158 genes (35.11%) were downregulated (Fig. 2; Table S2d). In addition, DEGs commonly regulated by both types of insect pests were also identified: 24 upregulated and 75 downregulated genes in leaves and 27 upregulated and 5 downregulated genes in sheaths (Fig. 2; Table S2e, f). For example, several defense-associated genes encoding Bowman-Birk trypsin inhibitors (Os01g0124200), peroxidases (Os03g0235000, Os10g0109600), BURP and universal stress protein A (UspA) domain-containing proteins (Os05g0217800, Os05g0217700, Os03g0305400) were commonly induced in leaves under separate leaffolder and BPH infestations (Table S2e).

**Fig. 3:**
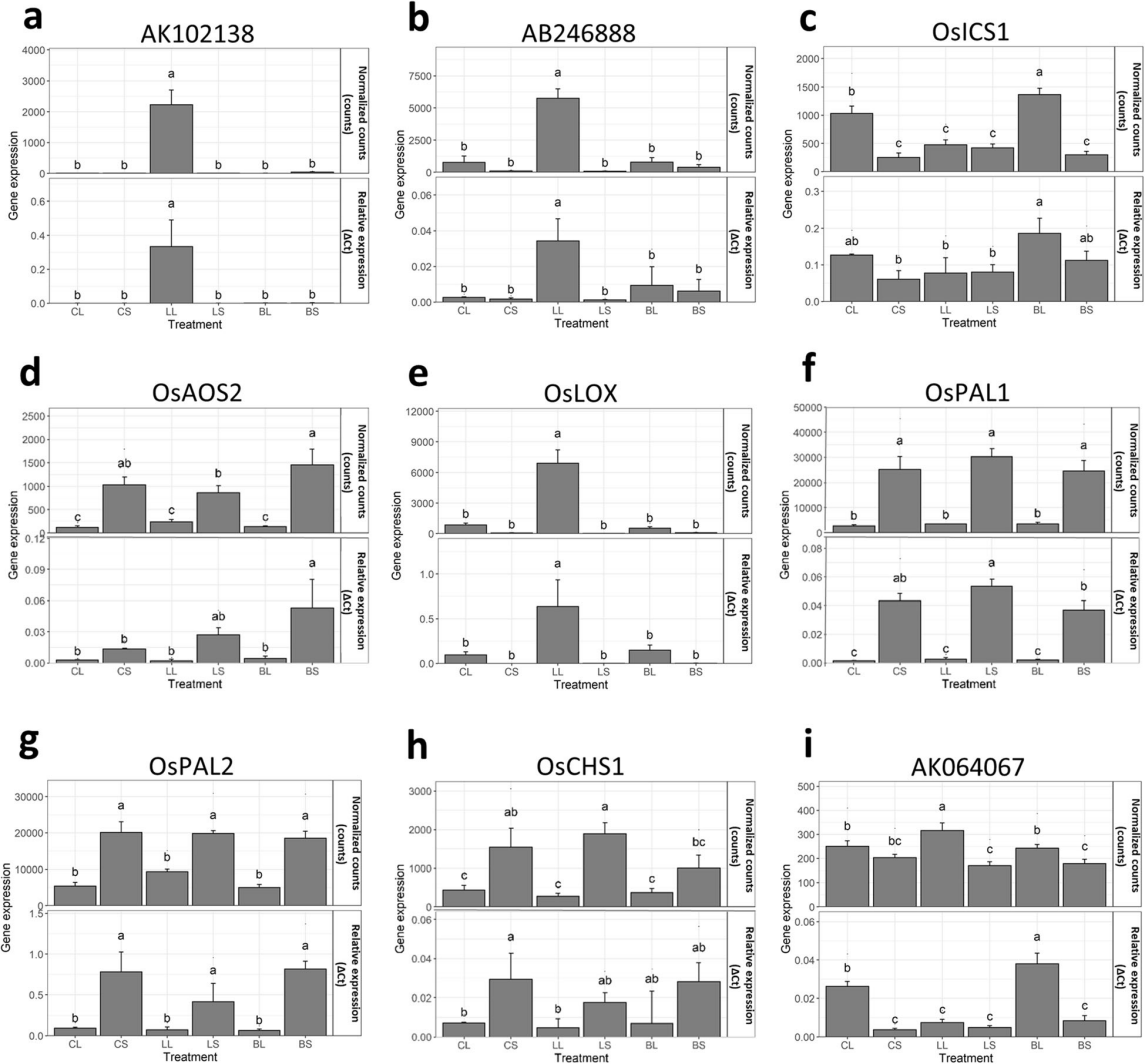
Validation of nine defense-related or sample-specific differentially expressed genes using qRT-PCR. For RNA-Seq, normalized counts of a gene in each sample (normalized counts ± SD) were measured in five biological replicates. For qRT-PCR, the relative gene expression in each sample (ΔCt ± SD) was measured in four biological replicates and three technical repeats with a ubiquitin gene (Os03g0234200) used as a reference gene. *OsICS1* = isochorismate synthase 1, *OsAOS2* = allene oxide synthase 2, *OsLOX* = lipoxygenase, *OsPAL1* = phenylalanine ammonia-lyase 1, *OsPAL2* = phenylalanine ammonia-lyase 2, *OsCHS1* = chalcone synthase 1, AK102138 = Bowman-Birk serine protease inhibitor family protein, expressed, AB246888 = NADPH oxidoreductase 1, AK064067 = conserved hypothetical protein, CL = control_leaf, CS = control_sheath, LL = leaffolder_leaf, LS = leaffolder_sheath, BL = BPH_leaf, BS=BPH_sheath. The measurements were analyzed with two-way ANOVA and LSD post hoc test with different letters denoting significant differences (*p* < 0.05)

Nine defense-related or sample-specific DEGs were shortlisted for qRT-PCR validation (Fig. 3). The qRT-PCR results of eight genes showed a strong correlation with the RNA-Seq results (except AK064067), attesting to the robustness of the generated transcriptomes.

### RNA-Seq reveals the involvement of JA biosynthesis and SA signaling in the Qingliu response to leaffolder herbivory and SA signaling against brown planthopper infestation

**Fig. 4:**
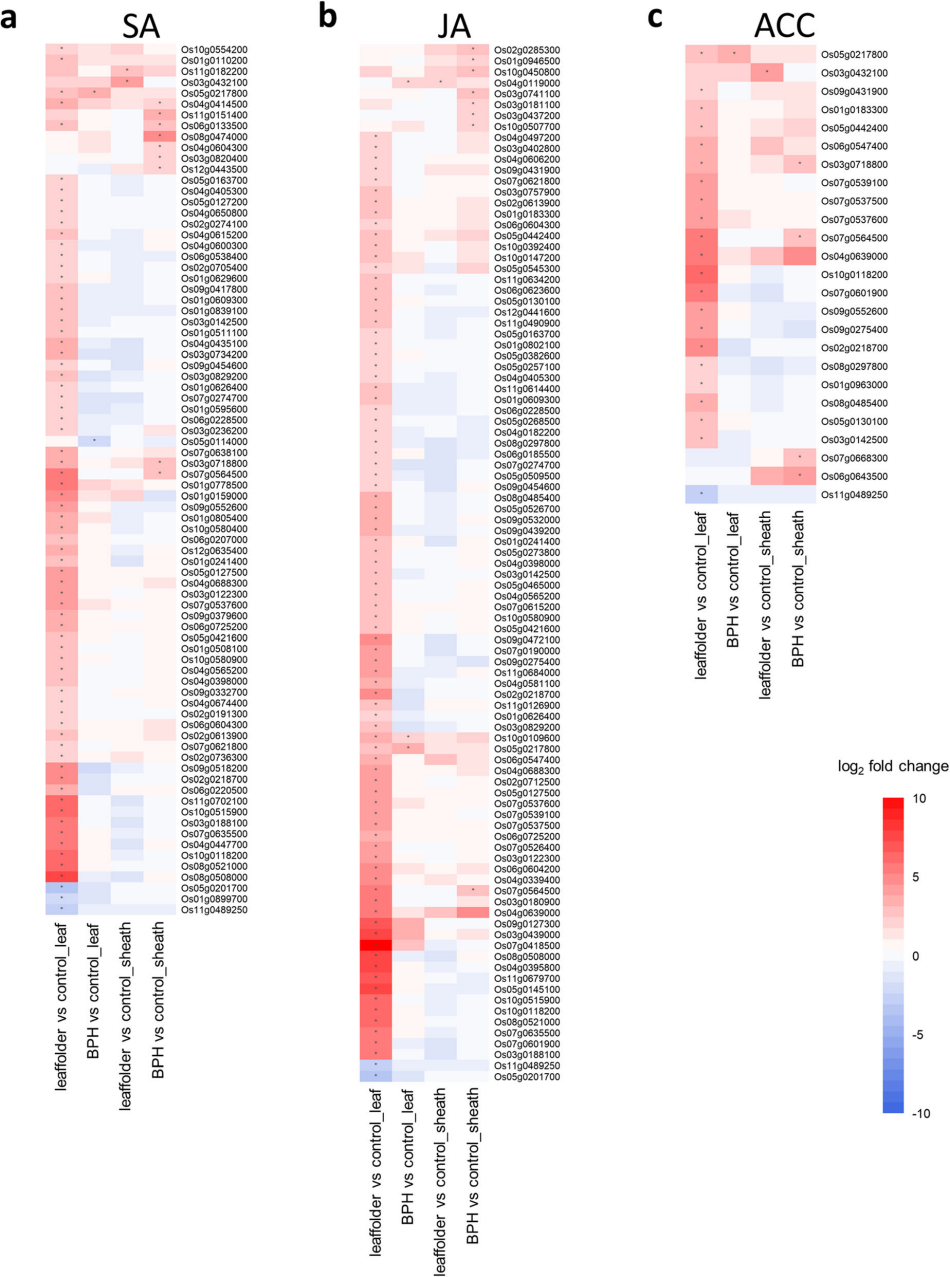
The expression patterns of defensive phytohormone-responsive genes in Qingliu under leaffolder and BPH infestation. **a** SA- **b** JA- and **c** ethylene- (ACC-) responsive genes identified in leaves and sheaths in response to infestation by insect pests. Red represents upregulation, and blue represents downregulation. DEGs with absolute log_2_-fold change > 1 and adjusted *p*-value < 0.05 are indicated with asterisks

To determine the defense phytohormones controlling Qingliu defense responses against either leaffolder or BPH infestation, the DEGs from plants infested with each insect were separately mapped to 798, 615 and 183 previously reported SA-, JA- and ethylene (ACC)-responsive genes, respectively [29]. A total of 80 SA-, 95 JA- and 25 ACC-responsive genes were mapped to the DEGs (Fig. 4; Table S3).

Notably, 86.3, 89.5 and 84.0% of the total mapped SA-, JA- and ACC-responsive genes, respectively, were induced by leaffolder feeding on the leaves, indicating the involvement of these three defense phytohormones in the modulation of the Qingliu defense response against this chewing insect (Fig. 4; Table S3). Induced expression of marker genes associated with the SA response were identified. These included abnormal inflorescence meristem 1 *OsAIM1* (Os02g0274100) that is involved in SA biosynthesis and two genes involved in SA signaling, nonexpressor of pathogenesis-related genes *OsNPR1/3* (Os03g667100 and Os01g0194300 (Table S2). For JA, although the expression of three JA biosynthesis genes was upregulated: lipoxygenases *OsLOX2.1/2.2/2.3* (Os08g0508800, Os12g0559200, Os08g0509100), seven genes encoding the repressors of JA signaling: jasmonate ZIM-domain proteins *OsJAZs* (Os03g0180900, Os10g0392400, Os07g0615200, Os03g0402800, Os09g0439200, Os03g0180800, Os04g0395800) were also upregulated (Table S2a). This result suggested the involvement of SA biosynthesis and signaling as well as JA biosynthesis in the defense response of Qingliu against leaffolder infestation.

Next, 11.3, 8.4 and 16% of the total mapped SA-, JA- and ACC-responsive genes, respectively, were induced in the BPH-infested sheaths (Fig. 4; Table S3). However, two genes encoding repressors of JA signaling, *OsJAZs* (Os03g0181100, Os03g0180800), were induced (Table S2d). This finding implicated the involvement of SA signaling in the defense response of Qingliu against BPH infestation. Indeed, among the nine SA-responsive genes upregulated in the BPH-infested sheaths, four genes were associated with transcription factor (Os08g0474000), cell wall modification (Os04g0604300, Os12g0443500) and lipid metabolism (Os03g0718800) (Fig. 4a; Table S3). In the coming sections, the relevance of these components in Qingliu defense response against the BPH will be explained based on Gene Ontology (GO) enrichment and MapMan annotation results.

### A defense response involving JA biosynthesis and the shikimate pathway is activated in the leaves of Qingliu plants upon leaffolder herbivory

To understand the defense response of Qingliu against leaffolder herbivory, GO enrichment analysis was performed separately on the up- and downregulated genes in each tissue. A higher number of enriched GO terms was found in the leaves (infestation site) than in the sheaths: upregulated genes in leaves were annotated to 68 GO terms, downregulated genes in leaves to 21 GO terms, upregulated genes in sheaths to 15 GO terms and downregulated genes in sheaths to 0 GO terms (Table S4). After removing the interrelated GO terms by manually curating the GO hierarchical tree graphs, upregulated genes in leaves were annotated to 22 GO terms, downregulated genes in leaves to 4 GO terms (mainly related to chlorophyll biosynthesis) and upregulated genes in sheaths to 1 GO term (Fig. S2).

**Fig. 5:**
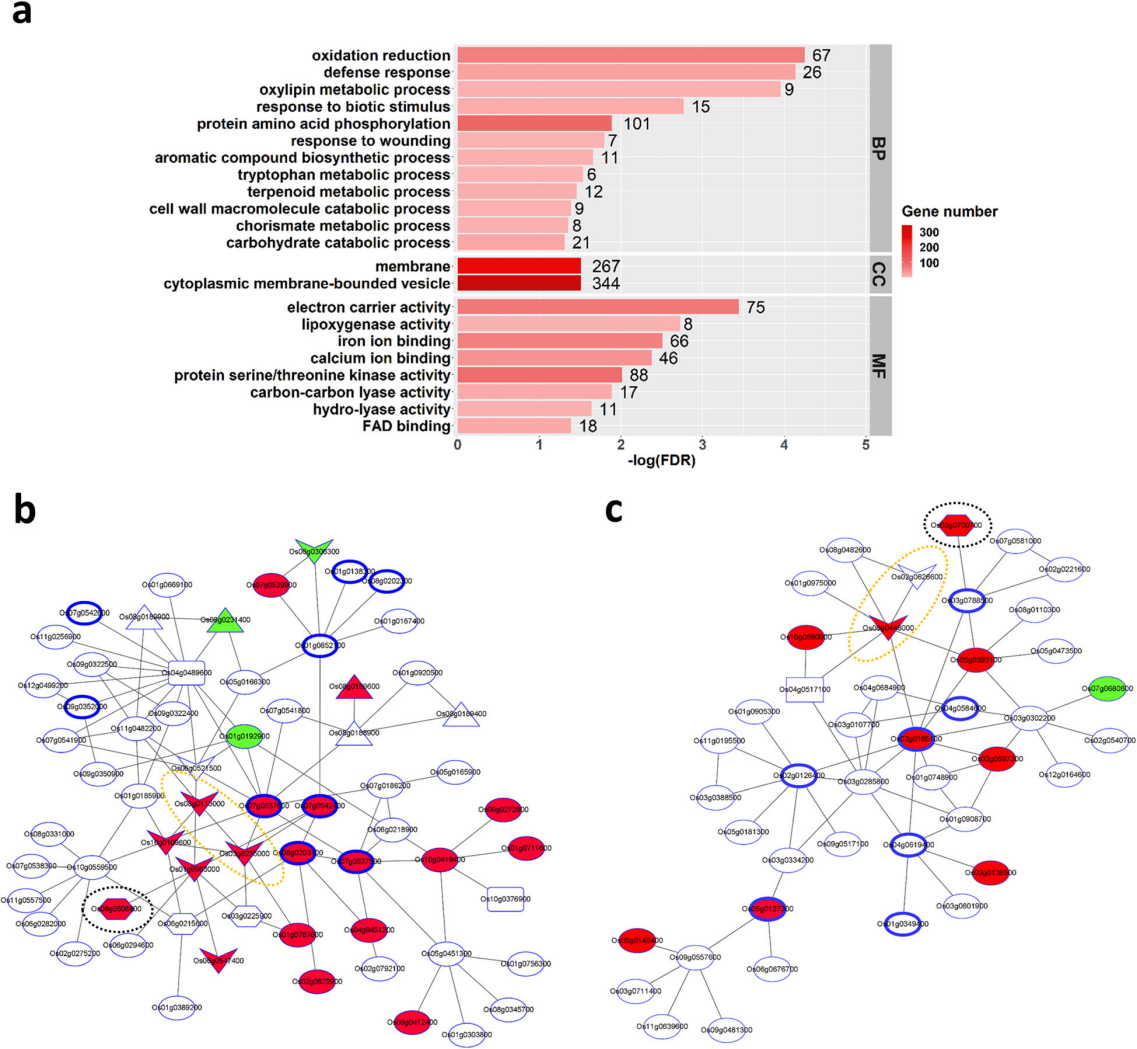
Determination of key defensive mechanisms taking place in Qingliu leaves under leaffolder herbivory. **a** GO enrichment of upregulated genes identified in Qingliu leaves in response to leaffolder herbivory. Enriched GO terms from biological process (BP), cellular component (CC) and molecular function (MF) GO categories are shown with bar length depicting enrichment significance and color intensity of bars depicting gene number. Coexpression network using **b** Os07g0542400, Os07g0537500, Os07g0537600, Os08g0203100 **c** Os02g0165100, Os05g0127300 coexpressed protein kinase genes induced by leaffolder feeding. Based on KEGG pathway annotation, genes associated with JA biosynthesis and the phenylpropanoid pathway are indicated by dotted line black and orange ellipses, respectively. Blue rimmed nodes = protein kinase genes, arrowhead nodes = genes encoding peroxidase, phenylalanine ammonia-lyase or 4-coumarate:coenzyme A ligase, hexagonal nodes = JA biosynthesis genes, square nodes = transcription factor genes, triangular nodes = germin-like protein genes. DEGs mapped to the coexpression networks are shown in red nodes for upregulation and green nodes for downregulation

The 22 enriched GO terms of upregulated genes in leaffolder-infested leaves consisted of 12 biological process terms, 2 cellular component terms and 8 molecular function terms (Fig. 5a; Table S4a). Based on the biological process annotation, it can be deduced that phosphorylation cascades (GO:0006468: protein amino acid phosphorylation) were activated in Qingliu as an early response to leaffolder infestation. The signaling cascades triggered JA biosynthesis and defense mechanisms in Qingliu (GO:0031407: oxylipin metabolic process, GO:0006952: defense response, GO:0009607: response to biotic stimulus, GO:0009611: response to wounding). Notably, the defense mechanisms involved an induction of the genes involved in cell wall catabolism, biosynthesis of aromatic amino acids (tryptophan, phenylalanine and tyrosine) via the shikimate pathway, and production of phenylpropanoid and terpenoid secondary metabolites (GO:0016998: cell wall macromolecule catabolic process, GO:0016052: carbohydrate catabolic process, GO:0019438: aromatic compound biosynthetic process, GO:0006568: tryptophan metabolic process, GO:0046417: chorismate metabolic process, GO:0006721: terpenoid metabolic process).

To demonstrate the role of phosphorylation cascades in activating the defense mechanisms of Qingliu against leaffolder infestation, coexpression analysis was performed using the 101 protein kinase genes annotated to GO:0006468: protein amino acid phosphorylation (Fig. 5a; Table S4a). At hierarchy = 0 and mutual rank (MR) = 14, four groups of coexpressed protein kinase genes were identified: (1) Os07g0542400, Os07g0537500, Os07g0537600, Os08g0203100; (2) Os02g0165100, Os05g0127300; (3) Os03g0773300, Os06g0602500; and (4) Os01g0664200, Os04g0514800. Hence, these four groups of coexpressed genes were separately used as guide genes to generate coexpression networks. At hierarchy = 2 and MR = 7, two biologically meaningful coexpression networks were generated from the first two groups of guide genes (Fig. 5b, c). The first coexpression network highlighted upregulated genes encoding (i) a lipoxygenase (Os08g0508800) involved in JA biosynthesis, and (ii) two peroxidases (Os03g0235000, Os08g0113000) annotated to three Kyoto Encyclopedia of Genes and Genomes (KEGG) pathways of osa01110: biosynthesis of secondary metabolites, osa00360: phenylalanine metabolism and osa00940: phenylpropanoid biosynthesis (Fig. 5b). The second coexpression network revealed induced expression of genes encoding another lipoxygenase (Os03g0700700) and a 4-coumarate:coenzyme A ligase (Os08g0448000) involved in lignin biosynthesis for defense against wounding (Fig. 5c). Notably, a phenylalanine ammonia-lyase gene (Os02g0626600) also was found in the coexpression network (Fig. 5c).

### A defense response involving cell wall modification and lipid metabolism is activated in the sheaths of Qingliu plants upon brown planthopper infestation

To decipher the defense response of Qingliu against BPH infestation, GO enrichment analysis was conducted separately on the up- and downregulated genes in each tissue. More enriched GO terms were discovered in the sheaths (infestation site) than in the leaves: upregulated genes in sheaths were annotated to 44 GO terms, downregulated genes in sheaths to 0 GO terms, upregulated genes in leaves to 2 GO terms and downregulated genes in leaves to 4 GO terms (Table S5). After removing the interrelated GO terms, upregulated genes in sheaths were annotated to 12 GO terms, upregulated genes in leaves to 1 GO term and downregulated genes in leaves to 1 GO term (Fig. S3).

**Fig. 6:**
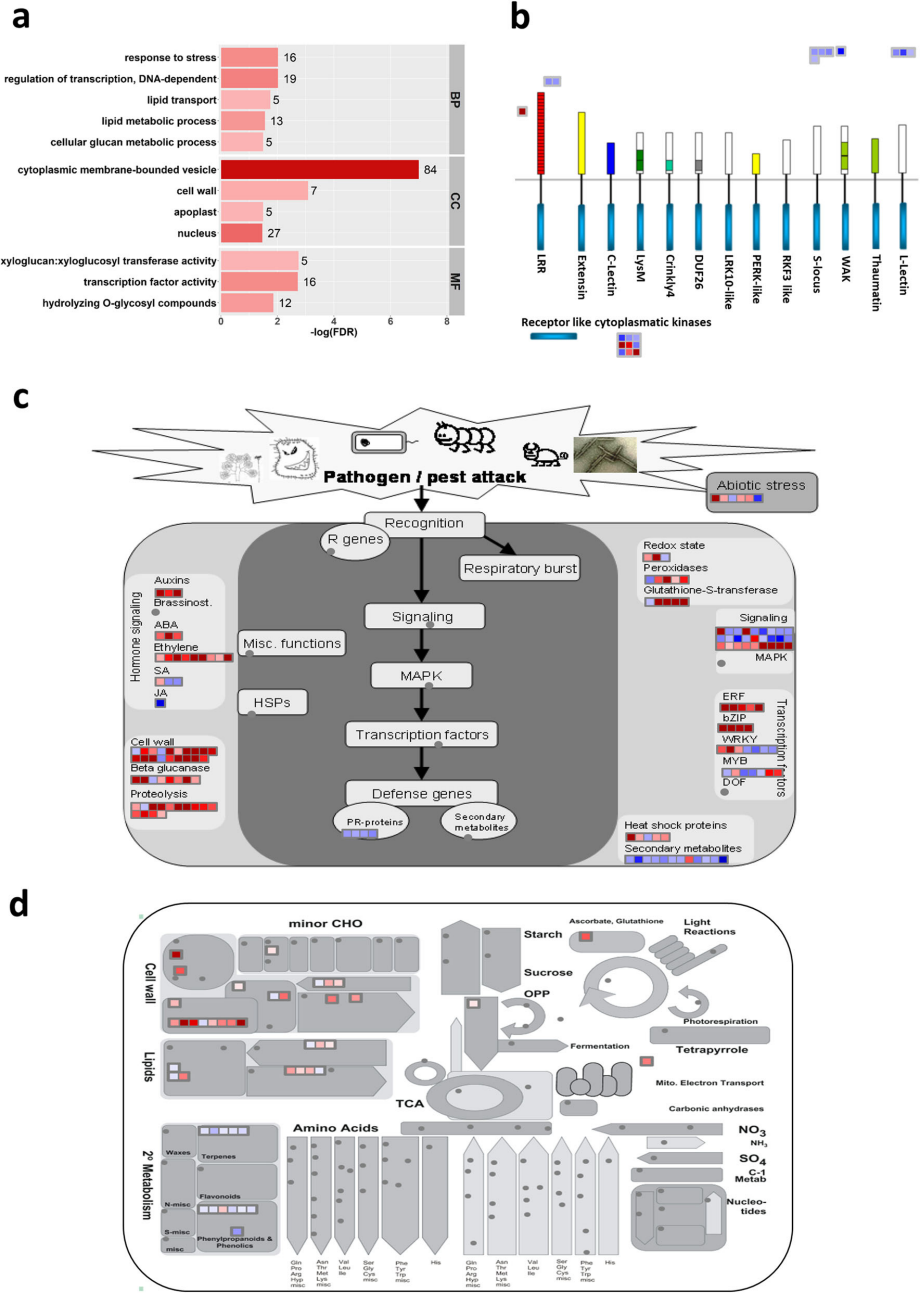
Determination of key defensive mechanisms taking place in Qingliu sheaths under BPH infestation. **a** GO enrichment of upregulated genes identified in Qingliu sheaths in response to BPH infestation. Enriched GO terms from biological process (BP), cellular component (CC) and molecular function (MF) GO categories are shown with bar length depicting enrichment significance and color intensity of bars depicting gene number. MapMan pathway annotation of DEGs with respect to **b** receptor kinases, **c** biotic stress and **d** overview of metabolism. Upregulated genes are shown in red and downregulated genes are shown in blue

The 12 enriched GO terms of upregulated genes in BPH-infested sheaths consisted of 5 biological process terms, 4 cellular component terms and 3 molecular function terms (Fig. 6a). The biological process annotation revealed that Qingliu responded to BPH infestation by undergoing transcriptional reprogramming (GO:0006355: regulation of transcription, DNA-dependent). This transcriptional modification activated defense mechanisms that regulate cell wall modification and lipid metabolism/transport (GO:0006950: response to stress, GO:0006073: cellular glucan metabolic process, GO:0006629: lipid metabolic process, GO:0006869: lipid transport). As expected, the molecular function and cellular component annotation supported the aforementioned defense response (GO:0003700: transcription factor activity, GO:0005634: nucleus, GO:0004553: hydrolase activity, GO:0016762: xyloglucan:xyloglucosyl transferase activity, GO:0005618: cell wall) (Fig. 6a; Table S5a).

To validate the defense response of Qingliu against BPH infestation shown by the GO enrichment, the DEGs in the sheaths were mapped to MapMan pathways (Fig. 6b-d; Table S6). As a result, eighteen genes encoding receptor kinases were responsive to BPH infestation with the following five genes being upregulated: Os01g0741200, Os01g0821900, Os02g0190500, Os03g0717000 and Os08g0374701 (Fig. 6b; Table S6a). The activated receptor kinases potentially trigger calcium, phophoinositide and G-protein signaling pathways that transmit signals to the nucleus via a series of protein phosphorylation events (Fig. 6c; Table S6b). In agreement with the GO enrichment results, this signal activates transcription factors (Fig. 6c; Table S6b) that control the expression of genes required for defense mechanisms, particularly cell wall modification and lipid metabolism (Fig. 6d; Table S6c).

## Discussion

### Dual resistance of Qingliu to leaffolders and brown planthoppers

Screening rice germplasm for dual resistance to chewing and phloem-feeding insect pests is an important goal in breeding programs aiming to generate broad-spectrum insect-resistant rice cultivars [12]. However, this effort could be hampered by the antagonistic roles of JA and SA in the mediation of rice defense mechanisms against chewing and phloem-feeding insects [23]. For example, previous studies reported that Baiqiaowan is resistant to leaffolders because it sustained less leaf roll damage and suppressed larval growth more than susceptible rice lines [24, 26]. Nevertheless, the SSST and MSST results in this study showed that Baiqiaowan is highly susceptible to BPH infestation and cannot be considered to have dual resistance (Table 1).

Previous studies consistently showed that Qingliu is resistant to leaffolder infestation [24–26]. This work began with natural insect infestation screening tests of 270 rice genotypes conducted at an experimental farm in Taiwan [24]. Among the plants tested, Qingliu had the lowest percentage of leaf roll damage [24]. A previous study showed that in both intact plants and detached leaves, third-instar leaffolder caterpillars that fed on Qingliu recorded a higher mortality rate and lower relative growth rate than those fed on the leaffolder-susceptible TN1 [26]. A number of unique attributes were previously identified in Qingliu, such as a high leaf trichome density, that could impede leaffolder infestation [25].

Although Qingliu is known to be a resistant to chewing insect pests, its resistance to phloem-feeding insect pests such as the BPH has not been reported. In this study, Qingliu was shown to be moderately resistant to the BPH (Fig. 1; Table 1). For example, based on the damage scores derived from the SSST and MSST experiments, Qingliu appeared to be moderately resistant to BPH infestation (Table 1). In the choice test, the piercing-sucking insect showed a lower settling preference for Qingliu than for TN1 (Fig. 1a). Furthermore, honeydew excretion was found to be lower for the individual BPHs feeding on Qingliu than TN1 (Fig. 1b).

This study showed that Qingliu displays a rarely observed dual resistance to leaffolder and BPH. However, according to the growth-defense tradeoff hypothesis, plant defense activation generally comes at the expense of plant growth due to limited metabolic resources that can be allocated either for growth or defense [30]. For example, growth inhibition by SA can be observed in constitutive defense mutants, which have a dwarf phenotype due to elevated SA accumulation or signaling [31]. Over the years it has been demonstrated that Qingliu has promising growth and yield performance under uninfested conditions [27], but it is unknown whether these indicators will drop drastically under infested conditions due to defense activation. Therefore, further phenotypic and genetic characterization of Qingliu is required prior to generating genetic markers for use in breeding programs aiming to generate broad-spectrum insect-resistant rice cultivars.

### The importance of shikimate and phenylpropanoid pathways in Qingliu-leaffolder interactions

The elicitors in the salivary secretions of leaf-chewing insects trigger host defenses via an intricate wound-signaling pathway in which JA biosynthesis plays an important role [18, 32]. This explains the the induction of lipoxygenases (JA biosynthesis genes; Os08g0508800, Os12g0559200, Os08g0509100) in the leaffolder-infested leaves (Table S2a). Although JA biosynthesis in rice plants increases under leaffolder herbivory, a previous study showed that Qingliu produces a higher level of JA than the leaffolder-susceptible TN1 [26]. JA biosynthesis was reported to increase rice resistance to leaffolders by activating the phenylpropanoid pathway [33]. Here, increased transcript levels were observed for biosynthesis genes in the shikimate pathway that produce three essential aromatic amino acids: tryptophan, phenylalanine and tyrosine (Fig. 5a; Table S4a). A recent comparative proteomic study showed that enzymes (Q10NY1, Q5VNW0) catalyzing the synthesis or chorismate, the precursor of aromatic amino acids were at higher levels in Qingliu than in TN1 [28]. In addition to being used for protein synthesis, these essential aromatic amino acids also serve as precursors of various defensive compounds, such as SA, polyphenolic compounds and lignin, via the phenylpropanoid pathway in plants [32, 34].

Tryptophan-derived secondary metabolites in plants generally include indole alkaloids, indole glucosinolates, camalexin, auxin, serotonin and melatonin [35]. In response to leaffolder herbivory, the induction of genes encoding biosynthetic enzymes for serotonin (Os08g0140300, Os08g0140500) and melatonin (Os08g0157500) was observed (Table S2a). A recent study showed that suppression of serotonin biosynthesis enhanced the resistance of rice to BPH and striped stem borers, presumably by elevating phenylalanine-derived SA levels [36]. Based on this finding, the observed increased biosynthesis of serotonin in response to leaffolder feeding might serve as a precursor for melatonin biosynthesis. Melatonin in plants delays senescence by enhancing the activity of antioxidant enzymes and increasing flavonoid biosynthesis [37].

Phenylalanine and tyrosine are channeled into the phenylpropanoid pathway to produce defensive secondary metabolites – flavonoids [38, 39]. Phenylalanine ammonia lyase (PAL) catalyzes the first and committed step in the phenylpropanoid pathway, synthesizing *trans*-cinnamate from phenylalanine [40]. The *trans*-cinnamate is then hydroxylated to form *p*-coumarate [41]. Recent studies indicate that the monocot grass family Poaceae contains a bifunctional phenylalanine/tyrosine ammonia lyase (PTAL), which can synthesize *p*-coumarate from tyrosine, bypassing the *trans*-cinnamate intermediate [35, 39]. In the RNA-Seq expression profile of Qingliu in response to leaffolder herbivory, the upregulation of genes encoding *OsPAL6* (Os04g0518400) was found together with the following biosynthesis enzymes for flavonoids: chalcone synthase (*CHS*; Os07g0526400), chalcone isomerase (*CHI*; Os06g0203600), chalcone reductase (Os04g0167800), flavanone 3-hydroxylase (Os03g0122300) and leucoanthocyanidin dioxygenase (Os01g0832600, Os03g0289800, Os05g0127500) (Table S2a). These results are supported by a recent comparative proteomic study, which showed that PAL, CHS and CHI were expressed at higher levels in Qingliu than in TN1 before and/or during infestation [28].

Lignin is also produced by the phenylalanine/tyrosine phenylpropanoid pathway in monocotyledonous plants [42, 43]. Given protective role of the plant cell wall, lignin biosynthesis and deposition can be triggered in response to wounding caused by chewing insect pests [44]. In the RNA-Seq results, the induction of genes encoding the biosynthesis enzymes for lignin monomers: 4-coumarate:CoA ligase (Os08g0448000, Os02g0177600), caffeic acid O-methyltransferase (Os08g0157500), caffeoyl CoA O-methyltransferase (Os08g0498400, Os08g0498100), coniferaldehyde 5-hydroxylase (Os10g0512400), cinnamyl alcohol dehydrogenase (Os02g0187800) and cinnamoyl-CoA reductase (Os08g0441500) was observed (Table S2a). The lignin monomers are eventually transported into the apoplast where they are polymerized by peroxidase and laccase [45]. In agreement with this finding, a study reported that the insect-specific toxin scorpion peptide LqhIT2 improved rice resistance to leaffolders by promoting JA biosynthesis, which in turn increased lignin deposition in the cell walls of leaf tissues [33].

The PAL pathway is the main route of SA biosynthesis in rice [46] and in this study the induction of marker genes associated with SA biosynthesis was found: *OsAIM1* (Os02g0274100) and SA signaling: *OsNPR1/3* (Os03g0667100, Os01g0194300) (Table S2a). A previous study showed that Qingliu has higher levels of SA at 6 h of leaffolder feeding, and the SA level in Qingliu is constitutively higher than that in leaffolder-susceptible TN1 prior to insect feeding [26]. The elevated SA biosynthesis in Qingliu under leaffolder herbivory might account for the observed suppressive effect on JA signaling due to the well-known antagonistic interaction between SA and JA [19]. This explains the paradoxical observation of the upregulation of genes involved in JA biosynthesis and negative regulators of JA signaling in Qingliu in response to leaffolder infestation (Table S2a). While the functional importance of SA biosynthesis and signaling is well-documented against phloem-feeding insect pests, the roles of SA against chewing insect pests still require further investigation [18, 47, 48]. Unless there is evidence suggesting currently unknown functions of SA signaling, the increased formation of SA through the PAL pathway might merely serve to repress JA signaling, whereas accumulation of phenylpropanoid-derived secondary metabolites mainly account for Qingliu resistance to leaffolders.

### The importance of cell wall modification and lipid metabolism in Qingliu-brown planthopper interactions

Compared with leaffolder larvae that cause extensive leaf damage, BPHs that insert their needle-like stylets through the apoplastic space between epidermal cells to reach sap-containing phloem cells cause little mechanical damage to host tissues [12]. By distinguishing the degree of tissue damage at the feeding site, rice plants are able to employ distinct defense mechanisms against these two types of insect pests with different feeding guilds [49]. To date, five BPH resistance loci have been functionally characterized by map-based gene cloning: *Bph3*, *Bph14*, *Bph18*, *Bph26* and *Bph29* [18, 47, 50–52]. Except for *Bph29*, which encodes a resistance gene containing a B3 DNA-binding domain (a potential transcription factor), the other four loci encode either coiled-coil, nucleotide-binding site and leucine-rich repeat proteins (CC-NBS-LRR) or lectin receptor kinases (LecRK), which activate the SA signaling pathway and induce callose deposition in phloem cells [18, 47, 50–52].

In this study, induced expression of five receptor kinase genes was found in BPH-infested sheaths at 24 h: Os01g0821900, Os08g0374701, Os03g0717000, Os02g0190500 and Os01g0741200 (Table S6a). Three of the receptor kinase genes have been reported as upstream regulators in signal transduction against abiotic and biotic stresses [53–55]. For example, rice receptor-like cytoplasmic kinase 253 (OsRLCK253; Os08g0374701) interacts with stress-associated proteins 1/11 (OsSAP1/11) in the plasma membrane, nuclear membrane and nucleus, and their overexpression in Arabidopsis improves water deficit and salinity tolerance [54]. An ortholog of rice lysin motif-type receptor-like kinase 4 (OsLysM-RLK4; Os01g0741200) in Arabidopsis triggers plant innate immunity against pathogens by functioning as a chitin recognition receptor to turn on the chitin signaling pathway [55].

Genes associated with calcium signaling (Os09g0482800, Os04g0492800, Os06g0683400, Os01g0949500, Os01g0955100), phosphoinositide lipid signaling (Os02g0285300) and G-protein signaling (Os02g0719000, Os05g0513800, Os05g0454200) were induced in BPH-infested sheaths at 24 h (Table S6b). These signaling pathways could lead to the activation of transcription factors in the nucleus. As expected, transcript levels of 28 genes mainly encoding transcription factors of the AP2/ERF (8 genes), MYB (5 genes), bZIP (3 genes), bHLH (3 genes) and WRKY (2 genes) families (Table S7a) were induced. Among the eight *AP2/ERF* transcription factor genes, four genes belonged to the *OsDREB* (dehydration response element binding) subfamily: *OsDREB1A*/*C*/*E*/*H*. These OsDREB transcription factors and OsbHLH148 are well known to control the rice response to drought, cold and salt stresses [56, 57]. Interestingly, the induction of a major *OsMYB4* transcription factor gene was observed, where a previous study comprehensively showed that the constitutive expression of *OsMYB4* in Arabidopsis improves tolerance to drought, salt, ultraviolet radiation, ozone, viruses, bacteria and fungi [58]. The induction of OsWRKY76 was also observed – its overexpression in rice plants was reported to increase susceptibility to *Magnaporthe oryzae* but enhance tolerance to cold stress [59].

Transcription factors reprogram the expression of genes controlling various downstream defense mechanisms in BPH-infested rice sheaths. The cell wall is the main “warzone” in the rice-BPH interaction; therefore, it is unsurprising that cell wall-related GO terms were enriched in the infested sheaths (Fig. 6) [60]. The salivary secretions of the BPH may contain cell wall modifying enzymes (CWMEs) that facilitate the penetration of stylets through the extracellular matrix of epidermal cells [61]. To counter this, rice plants also employ another set of CWMEs to fortify the cell wall, thwarting the feeding process of phloem sap-sucking pests [60]. Consistently, the expression of genes encoding enzymes essential for the biosynthesis, degradation and modification of major cell wall components, including pectin, cellulose, hemicellulose, and callose was induced in the BPH-infested rice sheaths at 24 h (Table S7b). For example, pectin lyase (Os12g0554800) catalyzes the depolymerization of pectin to oligogalacturonides, which function as damage-associated molecular patterns (DAMPs), activating the defense response through the mitogen-activated protein kinase (MAPK) signaling pathway [62, 63]. Xyloglucan, a hemicellulose, is a direct target degraded by pathogens for successful colonization of host plants [64]. To prevent the degradation of xyloglucan, the expression of four cell wall-localized xyloglucan endotransglucosylases/hydrolases (Os04g0604300, Os02g0696500, Os06g0696400, Os03g0239000), which serve to modify the xyloglucan chains, was induced in the BPH-infested sheaths of Qingliu (Table S7b) [64]. In addition, depolymerized xyloglucan oligomers can also function as DAMPs, triggering the MAPK signaling cascade [65]. Instead of callose synthase, induced expression of eight genes encoding β-1,3-glucanase was found, indicating that callose deposition in the phloem might not be the mechanism underlying the resistance of Qingliu to BPH (Table S7b) [66].

Lipid metabolism including lipid-mediated signaling and wax biosynthesis was another process regulated in BPH-infested rice sheaths (Table S7c). For lipid signaling, increased expression of genes encoding phospholipase C (Os02g0588500) and two phospholipase D enzymes (Os08g0401800, Os02g0120200) was found (Table S7c). Under environmental stress, phospholipase enzymes catalyze the hydrolysis of membrane phospholipids to generate signal messengers such as phosphatidic acid, diacylglycerol, phosphoinositides and inositol polyphosphates [67]. Increased expression of a fatty acid 2-hydroxylase gene (*OsFAH1*; Os12g0628400), which functions in the biosynthesis of sphingolipids, was also identified (Table S7c) [68]. Proper production of sphingolipids protects the integrity of plasma membrane microdomains required for reactive oxygen species generation through the Rac1-RbohB pathway, which confers rice immunity against *Magnaporthe oryzae* [68]. Intriguingly, two fatty acid elongase genes (Os02g0205500, Os05g0568000) were induced in the infested sheaths (Table S7c). In the endoplasmic reticulum, the fatty acid elongase complex catalyzes the elongation of C16 and C18 fatty acids to very-long-chain fatty acids (VLCFAs) with C26 to C34 chains [69, 70]. VLCFAs are converted to cuticular wax that forms the outermost hydrophobic layer of aerial organs for the protection of plants against abiotic and biotic stresses [71]. The cuticular wax produced in the endoplasmic reticulum needs to be transported to the cuticle [71]. Thus, increased expression of five genes encoding lipid transfer proteins (Os03g0718800, Os12g0114500, Os06g0643500, Os07g0175600, Os10g0505500) was identified (Table S7c).

## Conclusions

Dual resistance of rice to chewing and piercing-sucking insect pests is a rarely observed phenotype due to the involvement of different defense mechanisms in each type of resistance and the antagonistic crosstalk between these mechanisms. Here, Qingliu was reported to display dual resistance to leaffolder (chewing insect) and BPH (piercing-sucking insect). It is vital to discover the genetic loci in Qingliu that contribute to the dual insect resistance phenotype as this information will be useful for breeding programs aiming to generate broad spectrum insect-resistant rice cultivars. To achieve this objective, tissue-specific RNA-Seq profiling was carried out to decipher the key defense mechanisms of Qingliu under leaffolder and BPH infestation respectively. As a result, transcriptional activation of JA biosynthesis and the interconnected shikimate and phenylpropanoid pathways was found in the leaffolder-infested leaves of Qingliu, which may increase the production of various important defense-related secondary metabolites, including SA, melatonin, flavonoids and lignin. On the other hand, transcriptional activation of SA signaling and cellular signaling transduction was found in the BPH-infested sheaths of Qingliu, which may lead to cell wall modification and formation of cuticular wax that perturb BPH feeding. Although the defense responses of Qingliu to both types of insect pests seem largely unrelated, the phenylpropanoid pathway (or more specifically PAL) could be a possible upstream convergent route conferring dual resistance, which warrant further studies.

## Methods

### Plant materials

Seeds of the Qingliu rice variety were obtained from the Taichung District Agricultural Research and Extension Station, COA, Changhua, Taiwan; seeds of TN1 were obtained from the Department of Agronomy, National Taiwan University, and seeds of Mudgo, H105, TNG67 and Baiqiaowan were obtained from the Chiayi Agricultural Experiment Station, Taiwan Agricultural Research Institute. Qingliu, an indica rice variety, is resistant to *C. medinalis* [24]. The indica rice cultivar TN1 is highly susceptible to *C. medinalis* and *N. lugens* and was used as a susceptibility check [26, 72]. Rice seeds of Qingliu and TN1 were sterilized for 20 mins, rinsed in sterile water and sown on water-moistened filter paper in Petri dishes. After 2 days of incubation at 37 °C, germinated seeds were transferred to 600 ml beakers containing 1X Kimura B nutrient solution [73]. Seedlings were grown in a growth chamber set at 30/25 °C (day/night) with a 12/12 h (day/night) photoperiod. The 1X Kimura B nutrient solution was changed every 3 days. After 8–10 days (at approximately the two-leaf stage), seedlings of uniform size were transplanted individually into a plastic pot (64 mm diameter at the base, 95 mm diameter at the aperture, and height of 165 mm) containing rice paddy soil from Taoyuan, Taiwan, and grown in environmentally controlled growth chambers. Potted seedlings were treated with soluble fertilizer (33, 31, and 27 kg ha^− 1^ NPK, respectively) and watered every 2 days. Plants were used for experiments 30 days after germination.

### Insect colonies

#### Cnaphalocrocis medinalis

The *C. medinalis* colony was originally collected from the Taichung District Agricultural Research and Extension Station, COA, Changhua, Taiwan, and reared on maize seedlings (White Pearl, Known-You Seed Co., Taiwan) in mesh cages (BugDorm-4, MegaView, Taiwan) as described by Guo et al. 2019 [26]. The 3rd instar larvae were used in the experiments.

#### *Nilaparvata lugens*

The *N. lugens* biotype 1 colony was obtained from the Chiayi Agricultural Experiment Station, Taiwan Agricultural Research Institute, COA, Chiayi, Taiwan. *N. lugens* were reared on TN1 seedlings in mesh cages (BugDorm-4, MegaView, Taiwan) in a growth chamber set at 30/25 °C (day/night) with a 12/12 h (day/night) photoperiod.

### Standard seedbox screening test (SSST)

A SSST was used to evaluate the resistance of Qingliu to *N. lugens* [74]. Four varieties were used as BPH-resistant and BPH-susceptible checks in the SSST and the following modified seedbox screening test (MSST): Mudgo and H105 were BPH-moderately resistant checks [75]; TN1 and TNG67 were BPH-susceptible checks [72, 76], in addition to Baiqiaowan (a leaffolder-resistant check) [24]. A total of twenty-four seeds of each rice variety were sown in lines. Only twenty seedlings from each variety were selected to perform the SSST. At 14 days after sowing, seedlings were infested with 2nd- to 3rd-instar *N. lugens* nymphs at a density of 10 nymphs per seedling. The damage rating used was based on the standard evaluation system used for *N. lugens* infestation in rice, where 0 = no injury, 1 = slightly damaged, 3 = 1st and 2nd leaves of plants show yellowing symptoms, 5 = 10 to 25% of plants show pronounced yellowing and stunting or wilting symptoms, 7 = more than 50% of plants wilted, and 9 = all plants wilted or dead [77]. The resistance of rice lines was also determined based on the 0–9 scale where 0–3 was classified as resistant, 4–6 as moderately resistant and 7–9 as susceptible [74, 77]. This SSST experiment was repeated three times.

### Modified seedbox screening test (MSST)

The MSST experimental setup was modified from the standard evaluation system of the IRRI [77]. Briefly, six rice varieties were planted in a square plastic box (1 m × 1 m). In each box, each variety had four hills with 3–5 seedlings. At the tillering stage (ca. 30–35 days after sowing), rice plants were infested with gravid female *N. lugens* adults at a density of 0.5–1 adults per plant. When the susceptible checks (TN1) were moderately wilted due to *N. lugens* infestation, the condition of each seedling was scored using the same standard evaluation system as that used in the SSST every 2 to 3 days. This MSST experiment was repeated three times.

### Settlement preference test

Five germinated seeds of Qingliu and five germinated seeds of TN1 were transplanted to each plastic pot containing 1X Kimura B nutrient solution. Seeds of both varieties were transplanted on a metal platform in an alternating arrangement along the circumference of a 55 mm filter paper (ADVANTEC) with a 17 mm linear distance apart from one another. At 14 days after transplanting, 100 third instar *N. lugens* nymphs were released onto the filter paper of each hydroponic pot (a total of four pots). The pots were enclosed with plastic covers containing mesh cloth windows to prevent insects from escaping. The number of *N. lugens* nymphs settling on each rice variety was recorded at 3, 6, 24 and 48 h of infestation (*N* = 400).

### Honeydew excretion experiment

The honeydew excretion experiment using treated bromo-cresol green filter paper was adapted from Jena et al. (2017) [78]. Briefly, the 55 mm filter paper (ADVANTEC) was treated with 0.1% bromocresol green (Alfa Aesar) for 5 min. The tillers of rice plants except the major stem were removed before the experiment. A cardboard with a hole in the middle to accommodate the main stem was placed on each 30-day-old potted plant. Then, the dried bromo-cresol green filter paper was placed on the cardboard and covered by a plastic cup (22 mm diameter at the base, 45 mm diameter at the aperture, and height of 45 mm) (upside down) with a cotton plug to prevent *N. lugens* escape. One gravid female *N. lugens* starved for 1 h was released on each plant and allowed to feed. After feeding for 24 h, the filter paper was collected, scanned and analyzed using ImageJ [79]. Due to the chemical features of bromo-cresol green, blue-rimmed spots represent phloem-based honeydew, whereas white spots represent xylem-based honeydew. This experiment was repeated three times with a sample size of 8–10.

### Plant treatment, RNA isolation and sequencing library preparation

Each 30-day-old Qingliu rice plant was infested with fifteen 3rd instar BPH nymphs or one 3rd instar leaffolder larva for 24 h. The uninfested plants were used as controls. Each treatment (BPH infestation, leaffolder infestation and uninfested control) had 15 individual plants. Leaf and sheath tissues of each plant were collected separately and immediately frozen in liquid nitrogen and stored at − 80 °C. Total RNA was extracted with TRIzol reagent (Ambion) and purified using Quick-RNA Miniprep (Zymo Research) for each sample according to the manufacturer’s instructions. RNA integrity and quality were checked with a NanoDrop ND 1000 Spectrophotometer (Nanodrop Technologies, Wilmington, DE, USA). The total RNA of three individual plant tissues from the same treatment was pooled as one replicate. Each treatment had five biological replicates. Sequencing libraries were generated using the KAPA mRNA HyperPrep Kit (KAPA Biosystems, Roche, Basel, Switzerland) following the manufacturer’s recommendations. Library preparation and high-throughput RNA sequencing were performed by the Biotools Company (New Taipei City, Taiwan).

### Bioinformatics analysis

The original data obtained by high-throughput sequencing (Illumina NovaSeq 6000 platform) were transformed into raw sequenced reads by CASAVA base calling and stored in FASTQ format. FastQC and MultiQC were used to check the quality of fastq files [80]. The obtained raw paired-end reads were filtered by Trimmomatic (v0.38) to discard low-quality reads and trim adaptor sequences and eliminate poor-quality bases with the following parameters: LEADING:3 TRAILING:3 SLIDINGWINDOW:4:15 MINLEN:30 [81]. The obtained high-quality data (clean reads) were used for subsequent analysis. Read pairs from each sample were aligned to the rice reference genome (Os-Nipponbare-Reference-IRGSP-1.0) by HISAT2 software (v2.1.0) [82, 83]. FeatureCounts (v1.6.0) was used to count the read numbers mapped to individual genes [84]. For gene expression, “relative log expression” normalization (RLE) was performed using DESeq2 (v1.22.1) [85, 86]. DEG identification analysis between two conditions was performed in R using DESeq2. The resulting *p*-values were adjusted using Benjamini and Hochberg’s approach for controlling the false discovery rate (FDR) [87]. Genes that had an absolute log_2_-fold change higher than one (LFC1) and an adjusted *p*-value less than 0.05 were considered DEGs. GO enrichment and pathway analysis of DEGs were conducted with agriGO (v2.0) and MapMan (v3.6.0) with default settings [88, 89]. Gene coexpression analysis was performed using the RiceFREND server [90]. Phytohormone-responsive genes were extracted from Garg et al. (2012) [29], and heat maps to illustrate the expression change patterns were generated using the pheatmap package in R [91].

### Real-time RT-PCR

Total RNA samples from another independent experimental batch were used for qRT-PCR. For each sample, 1 μg of total RNA was reverse transcribed using the iScript cDNA Synthesis Kit (Bio-Rad, USA). Each qPCR consisted of 3 μL PCR-grade water, 5 μL iQ SYBR Green Supermix (Bio-Rad), 0.5 μL forward primer, 0.5 μL reverse primer and 1 μL cDNA template. qPCR was conducted on a CFX Connect Real-Time PCR Detection System (Bio-Rad) following the qPCR kit protocol. Melting curves were generated to confirm primer specificity. Relative expression of a gene of interest [ΔCt; ΔCt = Ct (gene of interest)-Ct (reference gene)] in each sample was measured in four biological replicates and three technical repeats. Primer details for nine genes of interest and a reference gene ubiquitin (Os03g0234200) are shown in Table S8. The reference gene ubiquitin (Os03g0234200) has been used as control for qPCR analysis in previous studies [92–94].

### Data analyses

Statistical analysis was carried out using the *z*-test or analysis of variance (ANOVA) as appropriate, and least significant difference (LSD) was used to test for differences between samples with alpha = 0.05. Data were analyzed using the free statistical software R (Version 3.5.1) [95].

## Supplementary Information

**Additional file 1: Fig. S1.** Principal component analysis (PCA) plot of thirty RNA-Seq samples. The samples consisted of three experimental conditions (control, BPH or leaffolder infestation) and two tissues (leaves, sheaths) with five biological replicates for each combination. **Fig. S2.** Shortlisting nonredundant GO terms of leaffolder-responsive DEGs by manually curating the GO hierarchical tree graphs. GO enrichment of **a** upregulated genes in the leaffolder vs control_leaf comparison, **b** downregulated genes in the leaffolder vs control_leaf comparison and **c** upregulated genes in the leaffolder vs control_sheath comparison. **Fig. S3.** Shortlisting nonredundant GO terms of BPH-responsive DEGs by manually curating the GO hierarchical tree graphs. GO enrichment of **a** upregulated genes in the BPH vs control_leaf comparison, **b** downregulated genes in the BPH vs control_leaf comparison and **c** upregulated genes in the BPH vs control_sheath comparison.

**Additional file 2: Table S1.** Statistics of raw reads and clean reads per RNA-Seq sample mapped to the rice reference genome.

**Additional file 3: Table S2.** List of differentially expressed genes. Up- and downregulated genes are shown for **a** leaffolder vs control_leaf comparison, **b** leaffolder vs control_sheath comparison, **c** BPH vs control_leaf comparison, **d** BPH vs control_sheath comparison, common DEGs in **e** leaves and **f** sheaths under separate leaffolder and BPH infestations.

**Additional file 4: Table S3.** The expression changes of defensive phytohormone-responsive genes in Qingliu under leaffolder and BPH infestations.

**Additional file 5: Table S4.** Details of GO enrichment results for the DEGs in Qingliu at 24 h of leaffolder herbivory. **a** Upregulated genes in the leaffolder vs control_leaf comparison, **b** downregulated genes in the leaffolder vs control_leaf comparison, **c** upregulated genes in the leaffolder vs control_sheath comparison and **d** downregulated genes in the leaffolder vs control_sheath comparison.

**Additional file 6: Table S5.** Details of GO enrichment results for the DEGs in Qingliu at 24 h of BPH infestation. **a** Upregulated genes in the BPH vs control_sheath comparison, **b** downregulated genes in the BPH vs control_sheath comparison, **c** upregulated genes in the BPH vs control_leaf comparison and **d** downregulated genes in the BPH vs control_leaf comparison.

**Additional file 7: Table S6.** MapMan pathway annotation results for the DEGs in the BPH-infested sheaths of Qingliu at 24 h. The mapping results of **a** receptor kinases, **b** biotic stress and **c** metabolism overview are shown.

**Additional file 8: Table S7.** Merged GO and MapMan results for the DEGs in the BPH-infested sheaths of Qingliu at 24 h. **a** transcription factor, **b** cell wall modification and **c** lipid metabolism.

**Additional file 9: Table S8.** Primers used for qRT-PCR amplifications.

## Data Availability

The RNA-Seq datasets generated and/or analyzed during the current study are available in the NCBI Sequence Read Archive (SRA) under accession number PRJNA689251 [https://www.ncbi.nlm.nih.gov/bioproject/PRJNA689251]. The reviewer link to the RNA-Seq datasets is provided [https://dataview.ncbi.nlm.nih.gov/object/PRJNA689251?reviewer=2b89mnmtv4988j74d7umd9hpq8] and the datasets will be released once the reviewers and Editor recommend acceptance, i.e. before the manuscript is accepted for publication. All data generated or analyzed during this study are included in this published article and its supplementary information files.

## Abbreviations

SSST: Standard seedbox screening test
MSST: Modified seedbox screening test
BPH: Brown planthopper
DEG: Differentially expressed gene
RNA-Seq: RNA sequencing
IPM: Integrated pest management
JA: Jasmonic acid
SA: Salicylic acid
ACC: Ethylene
QL: Qingliu
TN1: Taichung native 1
PCA: Principal component analysis
qRT-PCR: Real-time reverse transcription polymerase chain reaction
GO: Gene ontology
KEGG: Kyoto encyclopedia of genes and genomes
LOX: Lipoxygenase
COI: Coronatine-insensitive
AIM: Abnormal inflorescence meristem
NPR: Non expressor of pathogenesis-related
JAZ: Jasmonate ZIM-domain
PAL: Phenylalanine ammonia-lyase
CHS: Chalcone synthase
CHI: Chalcone isomerase
CC-NBS-LRR: Coiled-coil, nucleotide-binding site and leucine-rich repeat
CWME: Cell-wall modifying enzyme
DAMP: Damage-associated molecular pattern
MAPK: Mitogen-activated protein kinase
VLCFA: Very-long-chain fatty acid
FAH: Fatty acid 2-hydroxylase
