# Supplementary material for: Transcriptomics identifies key defense mechanisms in rice resistant to both leaf-feeding and phloem feeding herbivores
Source: BMC Plant Biol. 2021 Jun 30;21:306. doi: 10.1186/s12870-021-03068-5 (PMC8243607; doi:10.1186/s12870-021-03068-5)
Supplement: Supplementary file 1 — Additional file 1: Fig. S1. Principal component analysis (PCA) plot of thirty RNA-Seq samples. The samples consisted of three experimental conditions (control, BPH or leaffolder infestation) and two tissues (leaves, sheaths) with five biological replicates for each combination. Fig. S2. Shortlisting nonredundant GO terms of leaffolder-responsive DEGs by manually curating the GO hierarchical tree graphs. GO enrichment of a upregulated genes in the leaffolder vs control_leaf comparison, b downregulated genes in the leaffolder vs control_leaf comparison and c upregulated genes in the leaffolder vs control_sheath comparison. Fig. S3. Shortlisting nonredundant GO terms of BPH-responsive DEGs by manually curating the GO hierarchical tree graphs. GO enrichment of a upregulated genes in the BPH vs control_leaf comparison, b downregulated genes in the BPH vs control_leaf comparison and c upregulated genes in the BPH vs control_sheath comparison. [file 12870_2021_3068_MOESM1_ESM.pdf]

Supplementary Figures

Transcriptomics identifies key defense mechanisms in rice resistant to both leaf-feeding and phloem feeding herbivores

Yi Li, Boon Huat Cheah, Yu-Fu Fang, Yun-Hung Kuang, Shau-Ching Lin, Chung-Ta Liao, Shou-Horng Huang, Ya-Fen Lin, Wen-Po Chuang*

*Address for correspondence: Department of Agronomy, National Taiwan University, Taipei 10617, Taiwan; wenpo@ntu.edu.tw

Fig. S1

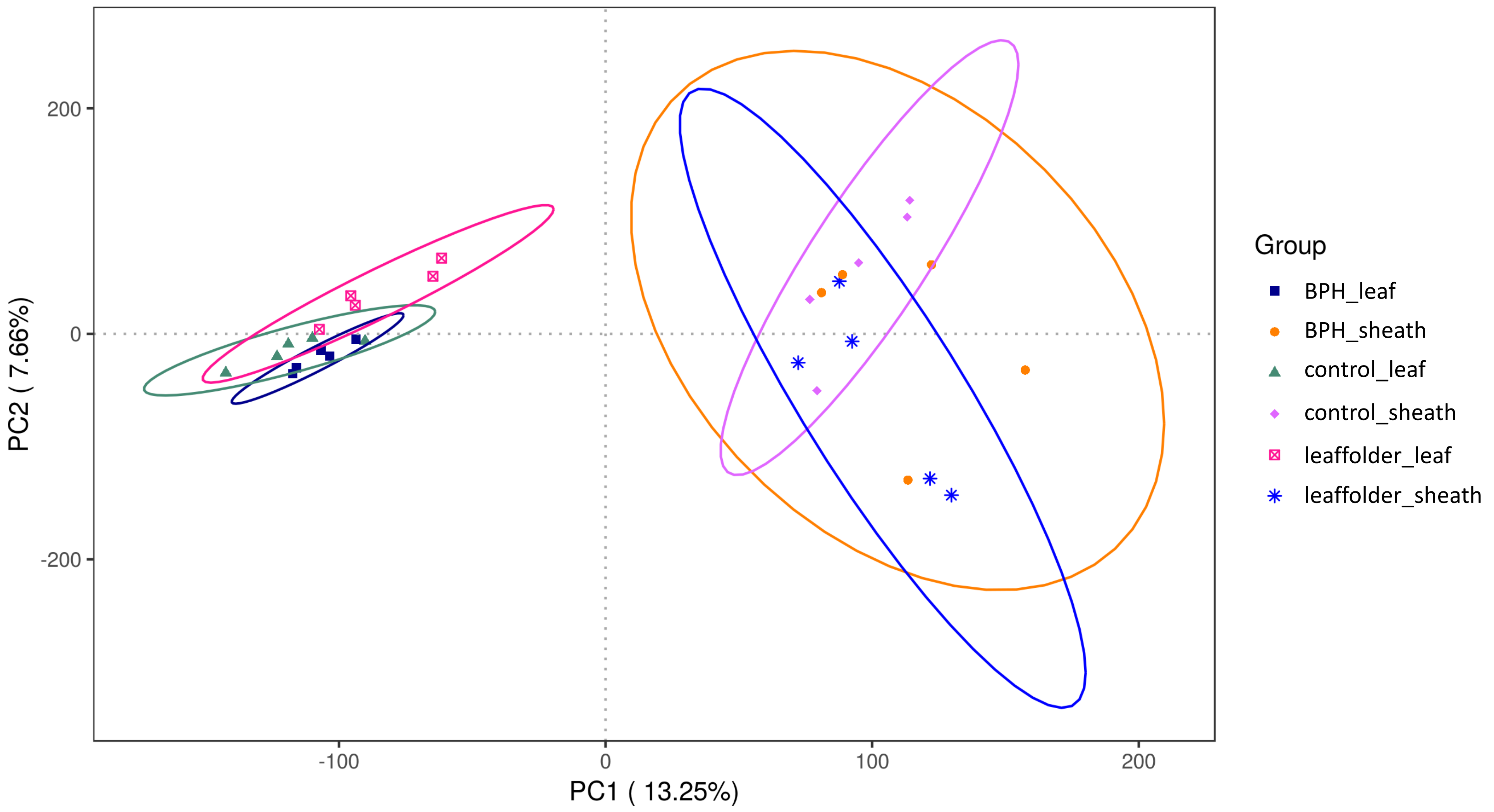

Fig. S1. Principal component analysis (PCA) plot of thirty RNA-Seq samples. The samples consisted of three experimental conditions (control, BPH or leaffolder infestation) and two tissues (leaves, sheaths) with five biological replicates for each combination.

Fig. S2

a. GO enrichment of upregulated genes in the leaffolder vs control_leaf comparison (Biological process)

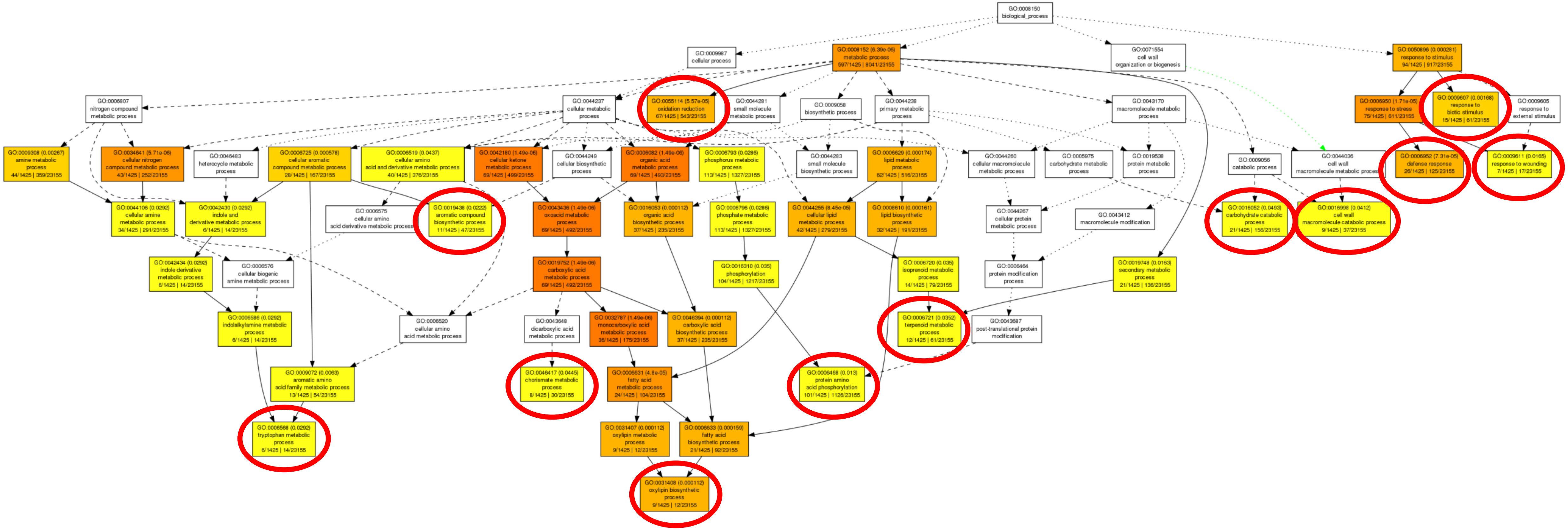

(Cellular component)

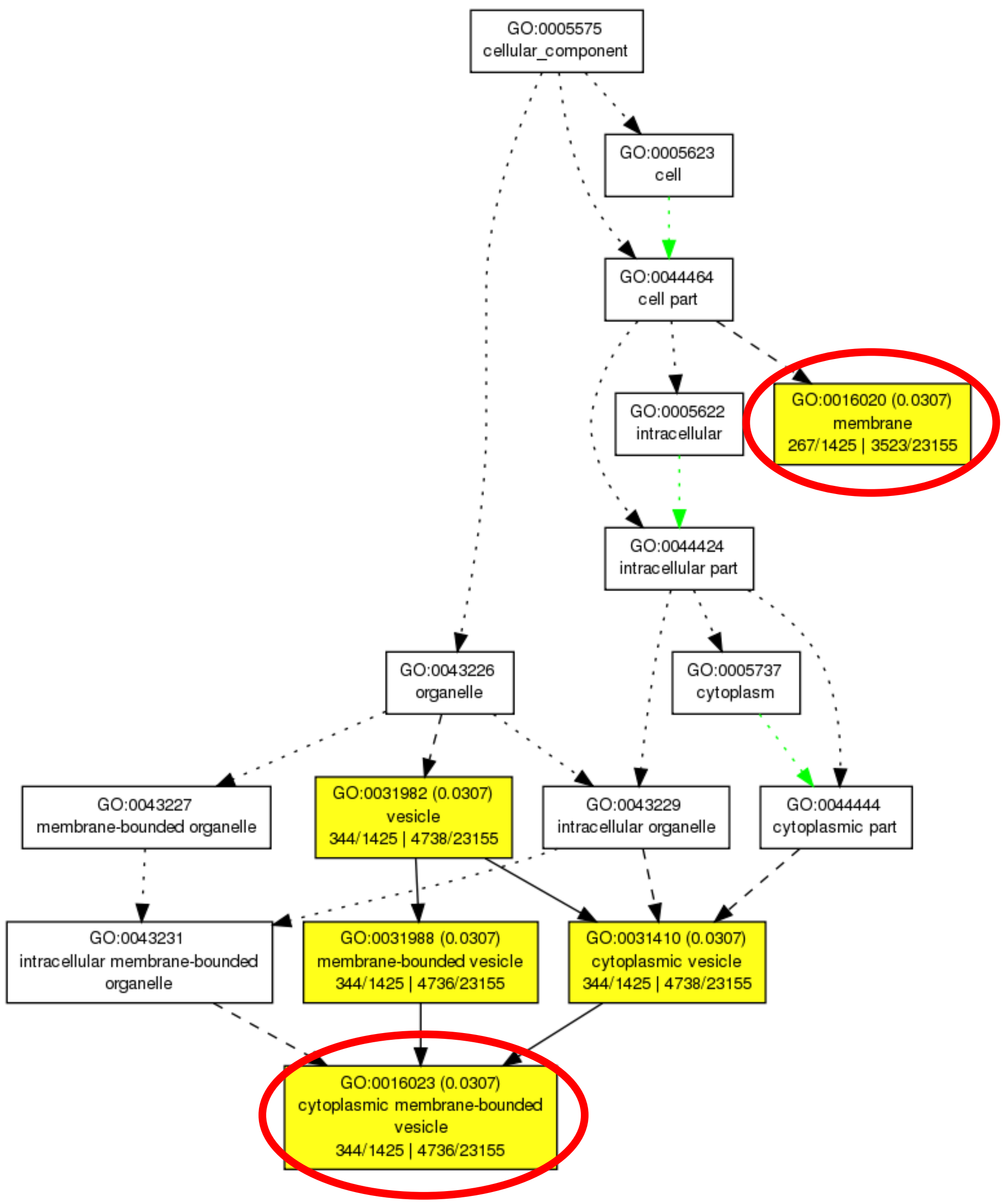

(Molecular function)

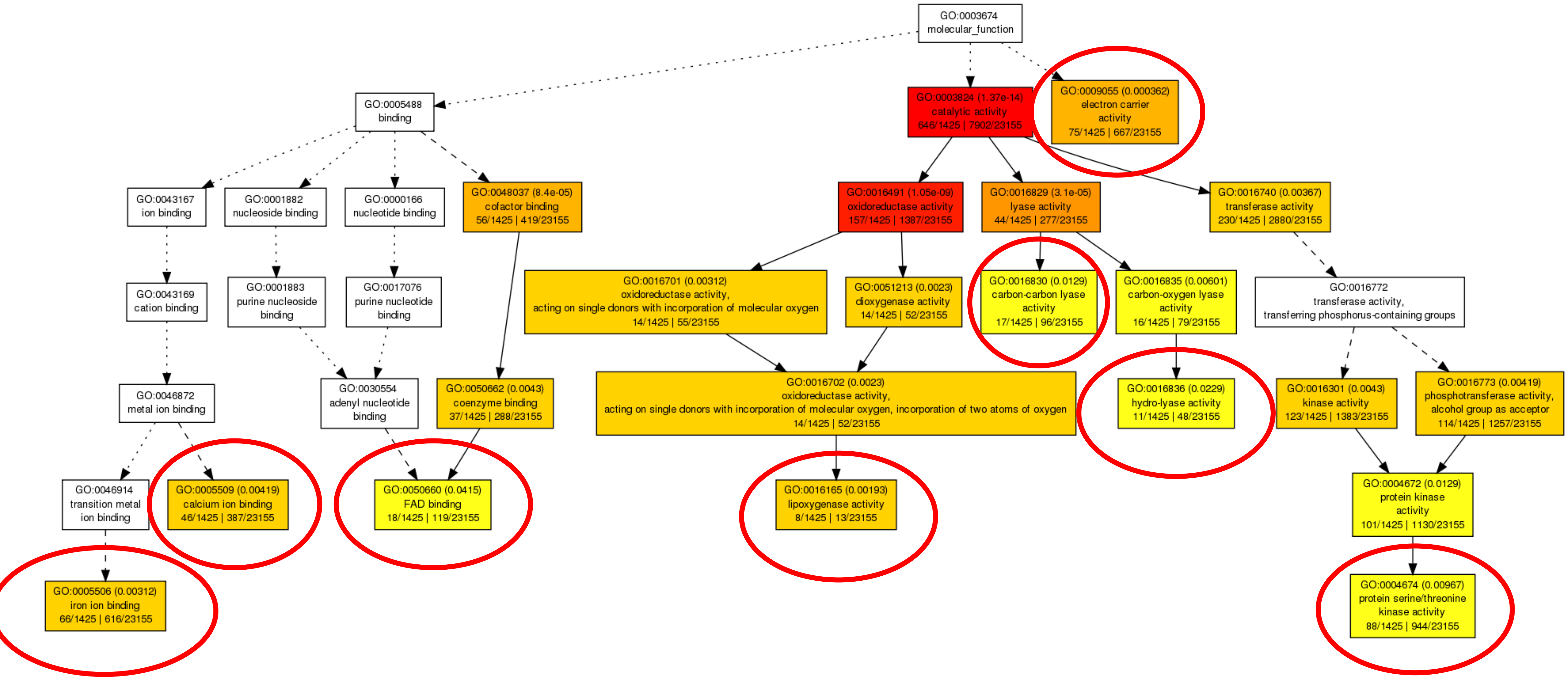

b. GO enrichment of downregulated genes in the leaffolder vs control_leaf comparison (Biological process)

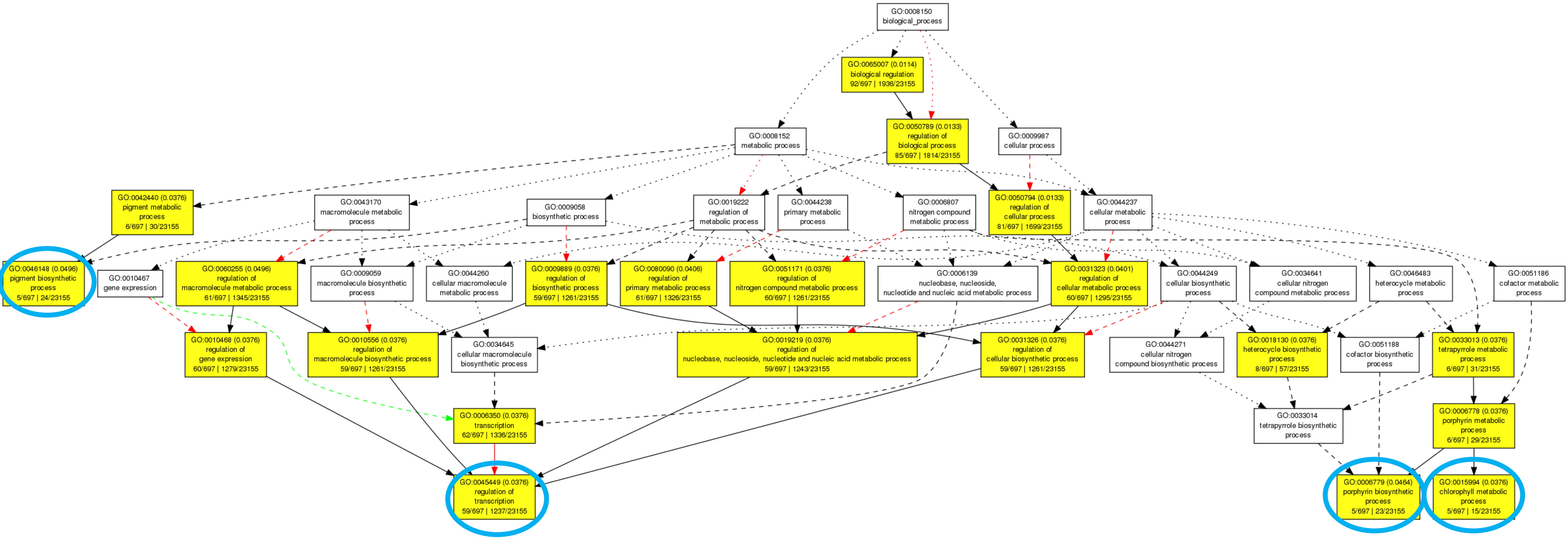

c. GO enrichment of upregulated genes in the leaffolder vs control_sheath comparison (Biological process)

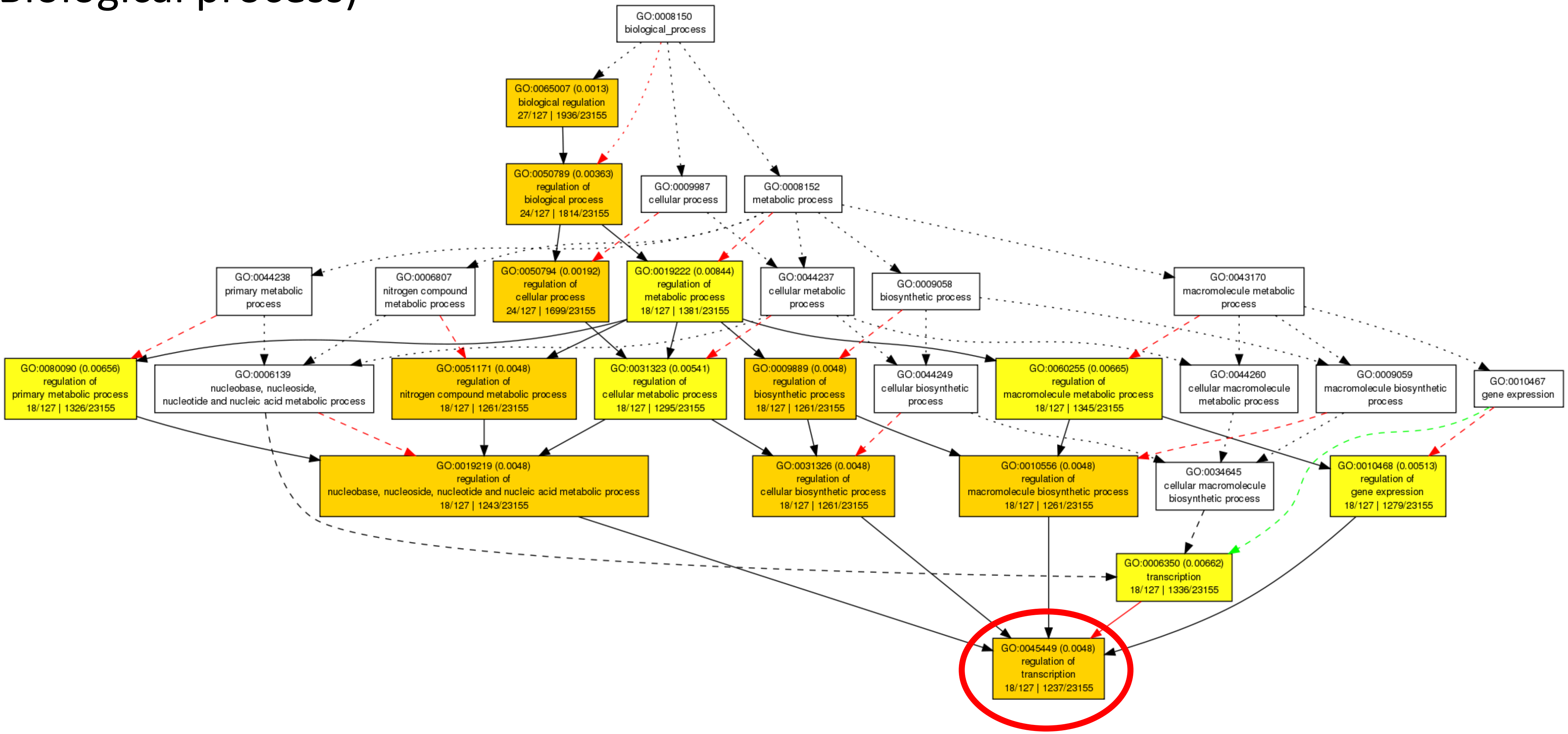

Fig. S2. Shortlisting nonredundant GO terms of leaffolder-responsive DEGs by manually curating the GO hierarchical tree graphs. GO enrichment of **a** upregulated genes in the leaffolder vs control_leaf comparison, **b** downregulated genes in the leaffolder vs control_leaf comparison and **c** upregulated genes in the leaffolder vs control_sheath comparison.

Fig. S3

a. GO enrichment of upregulated genes in the BPH vs control_leaf comparison (Cellular component)

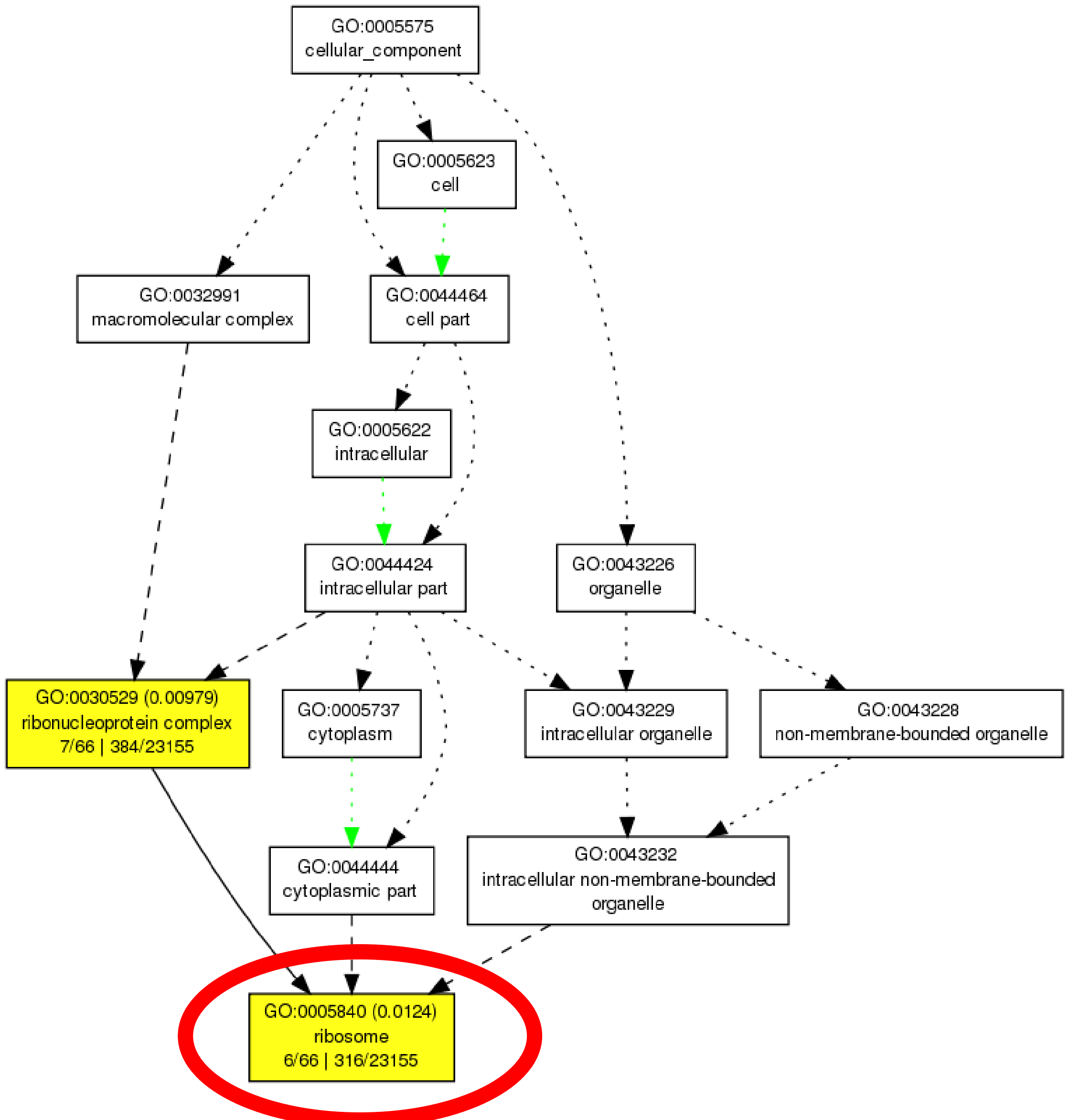

b. GO enrichment of downregulated genes in the BPH vs control_leaf comparison (Molecular function)

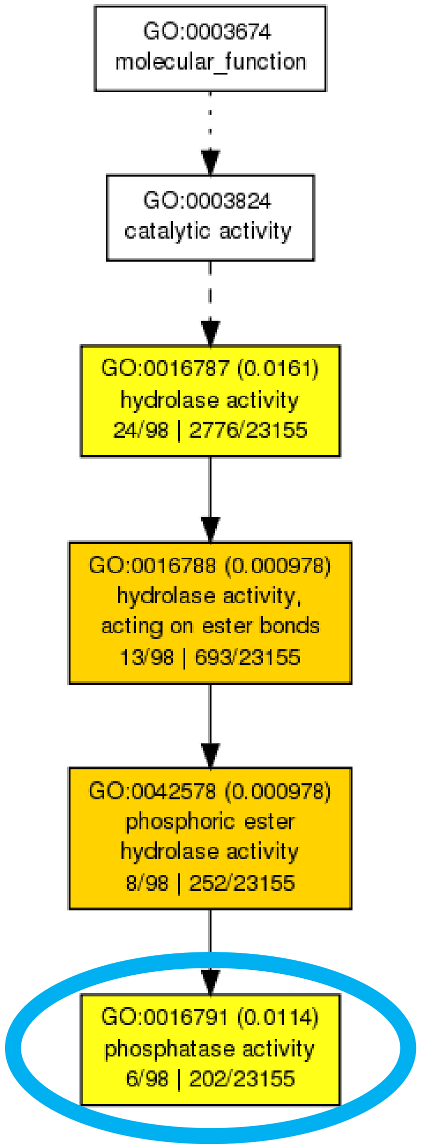

c. GO enrichment of upregulated genes in the BPH vs control_sheath comparison (Biological process)

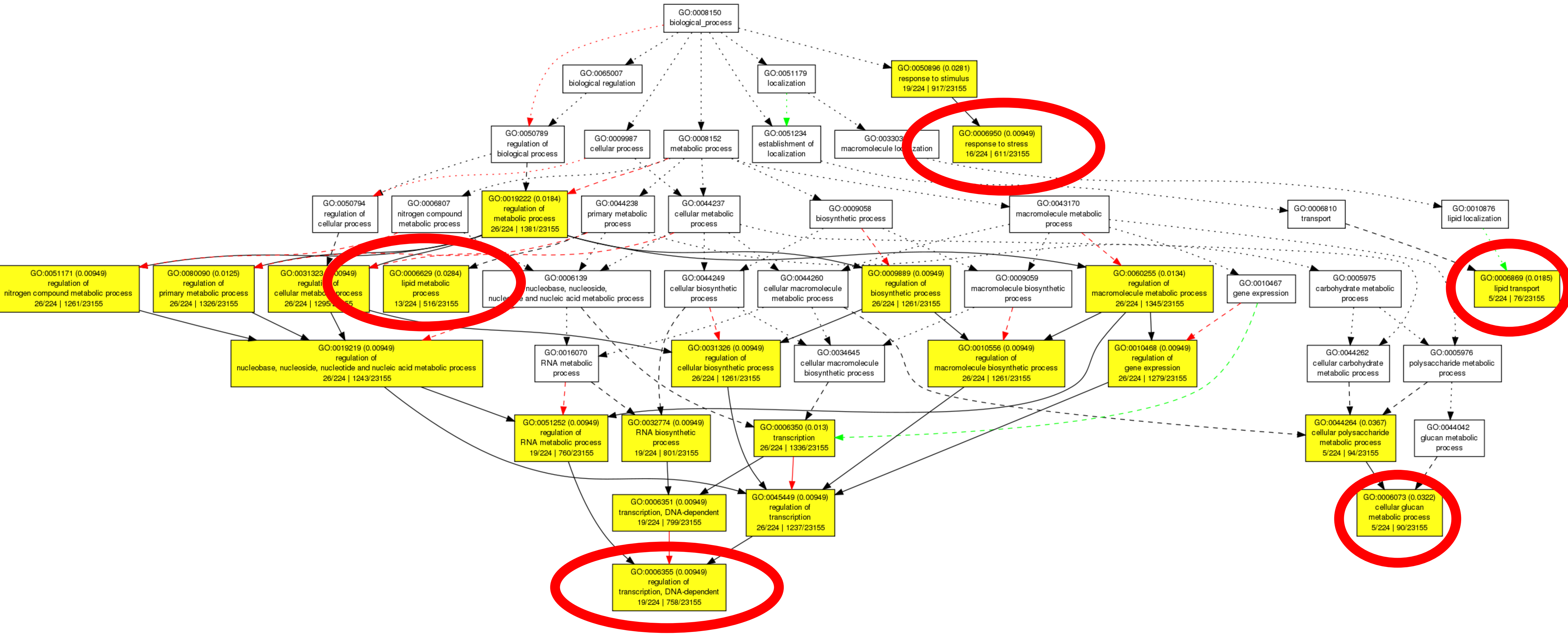

(Cellular component)

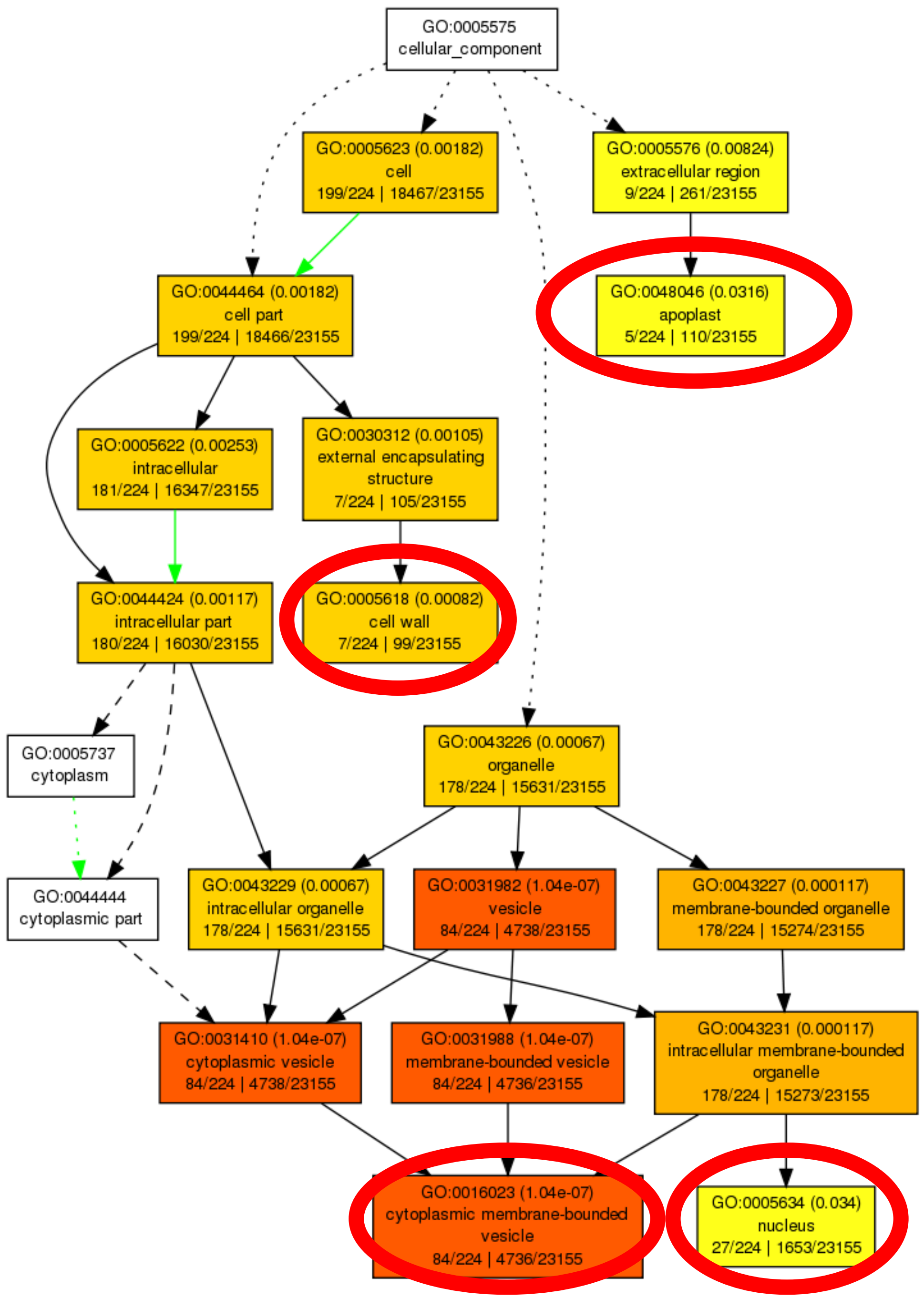

(Molecular function)

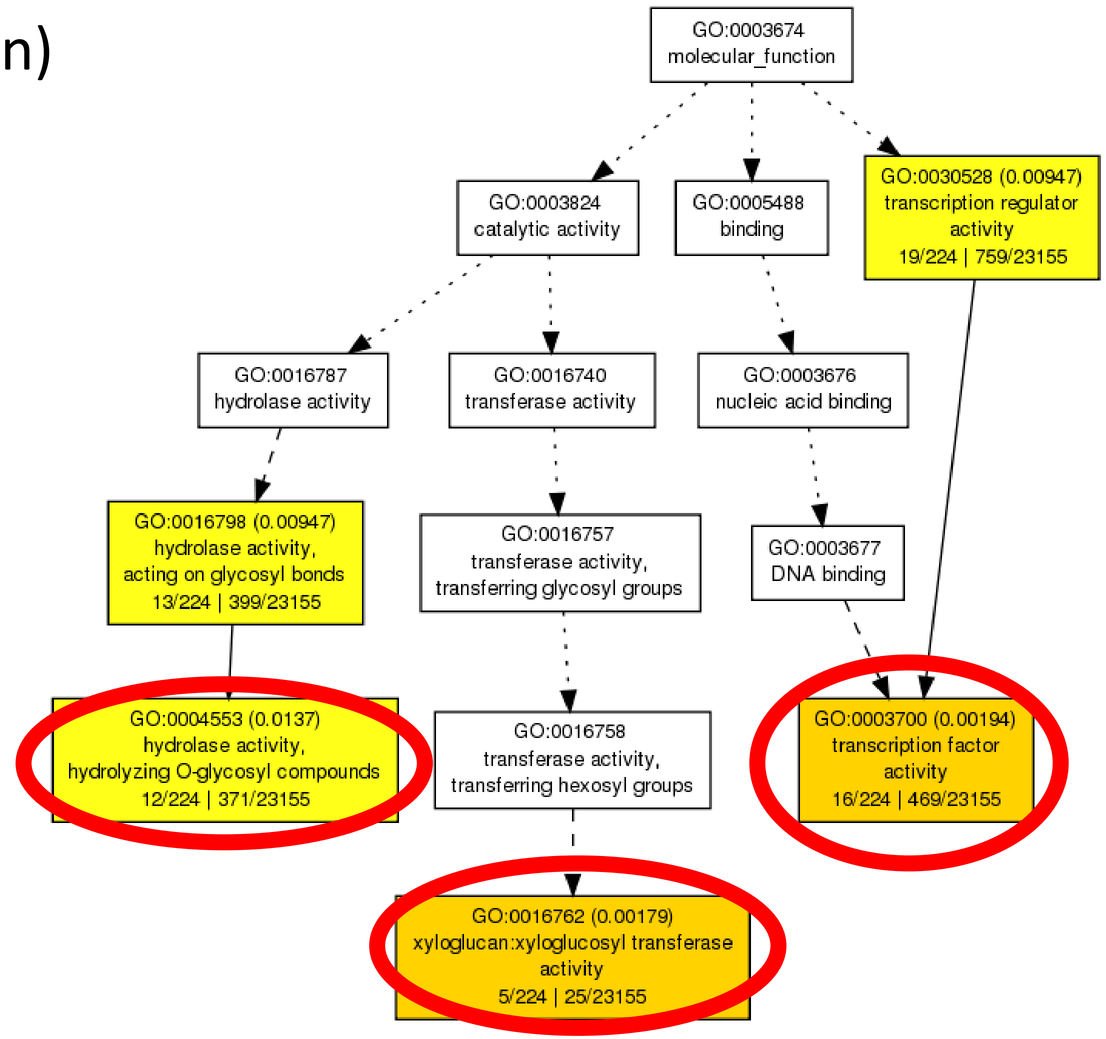

Fig. S3. Shortlisting nonredundant GO terms of BPH-responsive DEGs by manually curating the GO hierarchical tree graphs. GO enrichment of **a** upregulated genes in the BPH vs control_leaf comparison, **b** downregulated genes in the BPH vs control_leaf comparison and **c** upregulated genes in the BPH vs control_sheath comparison.
